# Optical imaging and spectroscopy for the study of the human brain: status report

**DOI:** 10.1117/1.NPh.9.S2.S24001

**Published:** 2022-08-30

**Authors:** Hasan Ayaz, Wesley B. Baker, Giles Blaney, David A. Boas, Heather Bortfeld, Kenneth Brady, Joshua Brake, Sabrina Brigadoi, Erin M. Buckley, Stefan A. Carp, Robert J. Cooper, Kyle R. Cowdrick, Joseph P. Culver, Ippeita Dan, Hamid Dehghani, Anna Devor, Turgut Durduran, Adam T. Eggebrecht, Lauren L. Emberson, Qianqian Fang, Sergio Fantini, Maria Angela Franceschini, Jonas B. Fischer, Judit Gervain, Joy Hirsch, Keum-Shik Hong, Roarke Horstmeyer, Jana M. Kainerstorfer, Tiffany S. Ko, Daniel J. Licht, Adam Liebert, Robert Luke, Jennifer M. Lynch, Jaume Mesquida, Rickson C. Mesquita, Noman Naseer, Sergio L. Novi, Felipe Orihuela-Espina, Thomas D. O’Sullivan, Darcy S. Peterka, Antonio Pifferi, Luca Pollonini, Angelo Sassaroli, João Ricardo Sato, Felix Scholkmann, Lorenzo Spinelli, Vivek J. Srinivasan, Keith St. Lawrence, Ilias Tachtsidis, Yunjie Tong, Alessandro Torricelli, Tara Urner, Heidrun Wabnitz, Martin Wolf, Ursula Wolf, Shiqi Xu, Changhuei Yang, Arjun G. Yodh, Meryem A. Yücel, Wenjun Zhou

**Affiliations:** a Drexel University, School of Biomedical Engineering, Science, and Health Systems, Philadelphia, Pennsylvania, United States; b Drexel University, College of Arts and Sciences, Department of Psychological and Brain Sciences, Philadelphia, Pennsylvania, United States; c Children’s Hospital of Philadelphia, Division of Neurology, Philadelphia, Pennsylvania, United States; d Perelman School of Medicine at the University of Pennsylvania, Philadelphia, Pennsylvania, United States; e Tufts University, Department of Biomedical Engineering, Medford, Massachusetts, United States; f Boston University Neurophotonics Center, Boston, Massachusetts, United States; g Boston University, College of Engineering, Department of Biomedical Engineering, Boston, Massachusetts, United States; h University of California, Merced, Departments of Psychological Sciences and Cognitive and Information Sciences, Merced, California, United States; i Lurie Children’s Hospital, Northwestern University Feinberg School of Medicine, Department of Anesthesiology, Chicago, Illinois, United States; j Harvey Mudd College, Department of Engineering, Claremont, California, United States; k University of Padua, Department of Developmental and Social Psychology, Padua, Italy; l Georgia Institute of Technology, Wallace H. Coulter Department of Biomedical Engineering, Atlanta, Georgia, United States; m Emory University School of Medicine, Department of Pediatrics, Atlanta, Georgia, United States; n Massachusetts General Hospital, Harvard Medical School, Athinoula A. Martinos Center for Biomedical Imaging, Charlestown, Massachusetts, United States; o University College London, Department of Medical Physics and Bioengineering, DOT-HUB, London, United Kingdom; p Washington University School of Medicine, Department of Radiology, St. Louis, Missouri, United States; q Chuo University, Faculty of Science and Engineering, Tokyo, Japan; r University of Birmingham, School of Computer Science, Birmingham, United Kingdom; s ICFO – The Institute of Photonic Sciences, The Barcelona Institute of Science and Technology, Castelldefels, Barcelona, Spain; t Institució Catalana de Recerca I Estudis Avançats (ICREA), Barcelona, Spain; u Washington University in St. Louis, Mallinckrodt Institute of Radiology, St. Louis, Missouri, United States; v University of British Columbia, Department of Psychology, Vancouver, British Columbia, Canada; w Northeastern University, Department of Bioengineering, Boston, Massachusetts, United States; x Université Paris Cité, CNRS, Integrative Neuroscience and Cognition Center, Paris, France; y Yale School of Medicine, Department of Psychiatry, Neuroscience, and Comparative Medicine, New Haven, Connecticut, United States; z University College London, Department of Medical Physics and Biomedical Engineering, London, United Kingdom; aa Pusan National University, School of Mechanical Engineering, Busan, Republic of Korea; ab Qingdao University, School of Automation, Institute for Future, Qingdao, China; ac Duke University, Department of Biomedical Engineering, Durham, North Carolina, United States; ad Duke University, Department of Electrical and Computer Engineering, Durham, North Carolina, United States; ae Duke University, Department of Physics, Durham, North Carolina, United States; af Carnegie Mellon University, Department of Biomedical Engineering, Pittsburgh, Pennsylvania, United States; ag Carnegie Mellon University, Neuroscience Institute, Pittsburgh, Pennsylvania, United States; ah Children’s Hospital of Philadelphia, Division of Cardiothoracic Anesthesiology, Philadelphia, Pennsylvania, United States; ai Polish Academy of Sciences, Nalecz Institute of Biocybernetics and Biomedical Engineering, Warsaw, Poland; aj Macquarie University, Department of Linguistics, Sydney, New South Wales, Australia; ak Macquarie University Hearing, Australia Hearing Hub, Sydney, New South Wales, Australia; al Parc Taulí Hospital Universitari, Critical Care Department, Sabadell, Spain; am University of Campinas, Institute of Physics, Campinas, São Paulo, Brazil; an Brazilian Institute of Neuroscience and Neurotechnology, Campinas, São Paulo, Brazil; ao Air University, Department of Mechatronics and Biomedical Engineering, Islamabad, Pakistan; ap Western University, Department of Physiology and Pharmacology, London, Ontario, Canada; aq University of Notre Dame, Department of Electrical Engineering, Notre Dame, Indiana, United States; ar Columbia University, Zuckerman Mind Brain Behaviour Institute, New York, United States; as Politecnico di Milano, Dipartimento di Fisica, Milan, Italy; at University of Houston, Department of Engineering Technology, Houston, Texas, United States; au Federal University of ABC, Center of Mathematics, Computing and Cognition, São Bernardo do Campo, São Paulo, Brazil; av University of Bern, Institute of Complementary and Integrative Medicine, Bern, Switzerland; aw University of Zurich, University Hospital Zurich, Department of Neonatology, Biomedical Optics Research Laboratory, Zürich, Switzerland; ax National Research Council (CNR), IFN – Institute for Photonics and Nanotechnologies, Milan, Italy; ay University of California Davis, Department of Biomedical Engineering, Davis, California, United States; az NYU Langone Health, Department of Ophthalmology, New York, New York, United States; ba NYU Langone Health, Department of Radiology, New York, New York, United States; bb Lawson Health Research Institute, Imaging Program, London, Ontario, Canada; bc Western University, Department of Medical Biophysics, London, Ontario, Canada; bd Purdue University, Weldon School of Biomedical Engineering, West Lafayette, Indiana, United States; be Physikalisch-Technische Bundesanstalt (PTB), Berlin, Germany; bf California Institute of Technology, Department of Electrical Engineering, Pasadena, California, United States; bg University of Pennsylvania, Department of Physics and Astronomy, Philadelphia, Pennsylvania, United States; bh China Jiliang University, College of Optical and Electronic Technology, Hangzhou, Zhejiang, China

**Keywords:** diffuse optics, optical spectroscopy, optical imaging, functional neuroscience, NIRS, DCS

## Abstract

This report is the second part of a comprehensive two-part series aimed at reviewing an extensive and diverse toolkit of novel methods to explore brain health and function. While the first report focused on neurophotonic tools mostly applicable to animal studies, here, we highlight optical spectroscopy and imaging methods relevant to noninvasive human brain studies. We outline current state-of-the-art technologies and software advances, explore the most recent impact of these technologies on neuroscience and clinical applications, identify the areas where innovation is needed, and provide an outlook for the future directions.

## 1 Introduction

We have come a long way since Jöbsis first demonstrated that noninvasive cerebral monitoring was possible in humans within the near-infrared light window (approximately 700 to 1000 nm) in the late 1970s.^1^ What started as a tool to quantify blood oxygenation in the brain has exploded into a whole field of study that aims to exploit this window to reveal a battery of information related to human brain physiology, including brain metabolism, blood flow, autoregulation, perfusion pressure, water concentration, light scattering, exogenous contrast agents, and more. In this text, we use the term “diffuse optics” to broadly describe any technique that employs a near-infrared light source and a photodetector to capture multiply scattered photons in the diffusion limit (*i.e.*, in the limit wherein the absorption coefficient, $\mu_{a}$, is much less than the reduced scattering coefficient, $\mu_{s}^{\prime }$, and the source-detector separation is much greater than the photon mean free path). The term “diffuse” has historical connotations in the field and reflects how near-infrared photons propagate through biological tissue. However, it should be noted that this term is in no way associated with how the field/community is organized or conducts its science. On the contrary, the breadth and depth of the progress made in the field over the past decade could only be accomplished by a cohesive, focused, and tight-knit community of researchers!

This review will focus primarily on two main diffuse optical techniques, near-infrared spectroscopy (NIRS) and diffuse correlation spectroscopy (DCS). In recent years, tremendous technological progress in both NIRS and DCS, along with advances in data analysis, have translated to improved accuracy, depth penetration, and spatial sensitivity. As a result, a multitude of applications to sense the brain in both health and disease have emerged over the past decade. Functional NIRS (fNIRS) has opened doors to explore unanswered questions in several fields, ranging from neurodevelopment to social and cognitive sciences to populations that are hard to assess with more conventional neuroimaging techniques like magnetic resonance imaging (MRI). (Note: In this report, we reserve the term “NIRS” for the techniques and their instrumentation, and the term “fNIRS" for experimental protocols that utilize diffuse optics to infer information about neural activity.) Moreover, the bedside monitoring capabilities of NIRS and DCS have led to their application in numerous clinical settings, ranging from neurocritical care to global health.

## 2 Hardware Developments

Technological improvements of diffuse optical instrumentation over the past decade have allowed more flexible and reliable data collection of oxygenation and blood flow in the human brain. In parallel, novel implementations of the physical ideas in multiply scattered light propagation have led to new data acquisition schemes with tremendous potential for improved depth penetration, spatial resolution, and signal-to-noise ratios. This section highlights the main technological advances across a range of instrumentation that employ near-infrared and correlation spectroscopies, along with the most updated efforts towards instrument standardization and commercialization.

### 2.1 Trends in Continuous-Wave NIRS (CW-NIRS)

The simplest and most widespread NIRS system employs continuous-wave light to illuminate the tissue. Commercial cerebral oximeters, which infer tissue oxygenation from intensity attenuation at the tissue surface, are the most well-known devices in the CW domain as they have gained clinical adoption for a variety of indications over the past decade. However, given their well-established commercial landscape, here we are primarily focused on CW-NIRS systems that measure temporal changes in the detected light intensity at the tissue surface. The intensity attenuation is attributed to changes in absorption and, by extension, to changes in the concentration of tissue chromophores, which are primarily oxy-hemoglobin (HbO) and deoxy-hemoglobin (HbR) in the brain. Standard CW-NIRS systems use laser diodes or light-emitting diodes (LEDs) as NIR sources to emit light, and photomultiplier tubes (PMTs) or avalanche photodiodes (APDs) to detect reflected, multiply-scattered light. The low cost and portability of CW-NIRS systems make this technique particularly well-suited for functional brain studies wherein neuronal activation elicits a pronounced hemodynamic response. While the general principles of CW-NIRS monitoring have remained unchanged for decades, recent advances in optical technologies and microelectronics have allowed for the advent of both high-density CW-NIRS systems that improve image quality, resolution, localization, and brain specificity, as well as wearable platforms that enable physiological monitoring in natural environments.

#### 2.1.1 High-density diffuse optical tomography (HD-DOT)

High-density diffuse optical tomography (HD-DOT) is an emerging approach for functional neuromonitoring that uses denser optode arrays than traditional CW-NIRS systems.^2^^–^^4^ HD-DOT provides numerous advantages over standard NIRS, including dramatically improved image quality, resolution, localization, and brain specificity. The density of the imaging arrays provides many crisscrossing measurements that enable tomography algorithms to aggregate measurements and partially deblur the diffuse propagation of signals through tissues. Further, different distances offer a limited ability for depth sectioning and flatter sensitivity to deeper depths (up to 2 cm from the scalp surface). When the imaging arrays are coupled with anatomically derived head models, the imaging becomes more compatible with fMRI style data analysis.^5^ When matched within-subjects against functional MRI (fMRI), HD-DOT can obtain localization errors <5  mm, and resolution <15  mm full width half maximum (FWHM), sufficient to localize functions to gyri.^5^ Feasibility studies have established HD-DOT for mapping sensory networks (visual and motor) as well as distributed cognitive networks, including the frontal, parietal, and default mode networks.^4^ A diverse set of functional neuroimaging paradigms has also been illustrated with HD-DOT, including traditional tasks,^2^ resting-state functional connectivity,^6^^,^^7^ naturalistic movie mapping,^8^ and most recently decoding studies.^9^

One of the main limitations of HD-DOT has been the mass of the fibers used in fiber-based HD-DOT systems. While fiber-based systems have thus far set the standard regarding specifications^2^^,^^6^^,^^10^ (including detectivity, dynamic range, crosstalk, frame rate, optode-scalp coupling, and modulation/demodulation strategies for encoding source illuminations^4^), several research groups have been demonstrating fiberless implementations of HD-DOT.^11^^–^^15^ Although technical issues are still to be worked out, we expect HD-DOT systems to transition to mostly fiberless implementations over the next five years, removing a main barrier to their use instead of sparse NIRS devices.

#### 2.1.2 Wearable CW-NIRS technology

Wearable CW-NIRS systems use lightweight and fiberless sources and detectors placed directly on the scalp, allowing for data acquisition in more ecologically valid environments.^14^^,^^16^ LEDs are the most widely used light sources, while APDs and silicon photodiodes are commonly used detectors in wearable CW-NIRS systems. Newer detectors, such as single-photon avalanche diode arrays (SPADs)^17^ and silicon photomultipliers, promise higher sensitivity than photodiodes.^18^ Recent wearable systems integrate digital conversion within the optodes, thereby eliminating the need for electrical cables to transfer the signal and making the design even more lightweight and compact.^15^^,^^19^ Moreover, a myriad of modular designs has been proposed that are flexible and adaptable to user needs.^15^^,^^19^^,^^20^

In the coming years, we anticipate that wearable systems will enable whole-head, high-density fNIRS measurements in everyday settings.^11^ In such settings, protection from ambient light and other environmental factors, robustness to motion artifacts, integration with multimodal measurements (e.g., eye tracking, motion sensors, visual/auditory input), and the synchronization of these additional measurements with the fNIRS signal will become more critical. Moreover, reliable data transmission and online detector sensitivity/signal quality control will be necessary. Early steps have been taken towards this end to extend Lab Streaming Layer (LSL) to support CW-NIRS data.^21^ LSL allows efficient and correct real-time transfer of data between computers and is commonly used in the brain-computer interface community. Its integration may also be used for broader application, such as real-time data quality management.

### 2.2 Frequency-Domain NIRS (FD-NIRS)

In contrast to the continuous-wave light sources used in CW-NIRS, frequency-domain near-infrared spectroscopy (FD-NIRS) uses intensity-modulated (>50  MHz) light sources.^22^^–^^26^ FD-NIRS is touted for its ability to separate tissue scattering from tissue absorption, yielding absolute measurements of $\mu_{a}(\lambda )$ and $\mu_{s}^{\prime }(\lambda )$ simultaneously. These absolute measurements can be valuable biomarkers of brain health and function, e.g., in the case of trauma or disorders of consciousness.^27^^,^^28^ Moreover, knowledge of the absolute optical properties is critical for accurately estimating the differential path length factor, which provides a more accurate estimation of depth and volume sensitivity of fNIRS data. FD-NIRS amplitude at typical modulation frequencies (100–150 MHz) has a similar sensitivity to changes in absorption and scattering as CW-NIRS. However, a primary benefit of FD-NIRS is that the measured phase difference between the incoming light and scattered light provides higher sensitivity and depth than CW-NIRS.^24^ FD-NIRS phase has also been used to identify fast optical scattering-driven evoked-response optical signals (EROS) that may be indicative of neural activity.^29^^,^^30^ Finally, when used for DOT, FD-NIRS enables higher resolution and better localization of the activation regions than CW-NIRS.^31^

Currently, the main limitations of FD-NIRS are its limited accessibility (e.g., as of now, only one company sells a FD-NIRS system), higher cost, larger footprint, and higher noise compared to CW-NIRS. Recent technology developments have begun to overcome these limitations and increase performance. Digital modulation and/or detection schemes have led to significantly faster (>1  kHz) and compact FD-NIRS systems.^32^^–^^36^ Creating new, application-specific integrated circuits for FD-NIRS modulation/demodulation,^37^ combined with vertical-cavity surface-emitting lasers^38^^,^^39^ and silicon photomultiplier detectors^40^^,^^41^ could lead to ultracompact, wearable, and higher signal-to-noise ratio (SNR) FD-NIRS sensors.

Given the recent renewed interest in FD-NIRS technology development and the formation of at least one new startup that aims to bring more FD-NIRS to the market, we expect this technology to become more accessible both in terms of cost and availability. As these developments happen, we expect that the advantages of the FD approach will be utilized by researchers in both existing and new applications.

### 2.3 Time-Domain NIRS (TD-NIRS)

In TD-NIRS measurements, the photon distribution of time-of-flight (DTOF) is recorded with picosecond resolution. Because the DTOF is sensitive to the optical properties of the sampled medium, the absolute values of $\mu_{a}$ and $\mu_{s}^{\prime }$ can be estimated in homogeneous and heterogeneous structures (e.g., two-layer medium, medium with embedded inhomogeneity) using physical models for photon migration in a diffusive medium.^42^ Further, through semi-empirical approaches (i.e., time windows, moments),^43^ it is possible to enhance depth discrimination of absorption changes, thereby enabling the rejection of extracerebral effects in fNIRS applications in an easier way than the CW-NIRS^44^ (see Sec. 3.2.4).

Recent developments in TD-NIRS instrumentation embrace the use of advanced photonic and microelectronic components (e.g., pulsed laser on a chip, single-photon detectors, time-correlated single photon counting electronics). These novel technologies empower the realization of compact, portable, and rugged commercial TD-NIRS systems,^45^^,^^46^ reducing the gap with CW- and FD-NIRS systems, which are typically smaller, lighter, and less expensive than TD-NIRS. The miniaturization of TD-NIRS components at the research level also comprises fast gating capabilities embedded in ultrasound probes for improved performance.^47^

Continued technological advances will bring wearable, high density, and miniaturized TD-NIRS apt for consumer applications and/or for multimodal integration (e.g., with EEG/EMG for brain/muscle applications) at the research and clinical levels. We also foresee improvements in TD-NIRS data analysis thanks to artificial intelligence tools that will handle the larger volume of data available in TD-NIRS.^48^

### 2.4 Hyperspectral and Broadband NIRS

Most NIRS instruments use two near-infrared wavelengths, which is the minimum requirement for quantifying HbO and HbR. The hyperspectral regime expands acquisition from two/few wavelengths to tens or hundreds of sequential wavelengths measured in a 2D image as a function of time, creating an extensive three-dimensional dataset often referred to as hypercube.^49^ A broadband spectrum is created when one collapses the hypercube to a time and spectral dimension. Broadband NIRS (bNIRS) is the acquisition of a full NIR spectrum consisting of hundreds of wavelengths at every time sample,^50^ which provides: (1) extended capacity in resolving chromophores beyond oxy- and deoxy-hemoglobin, such as the changes in cytochrome-c-oxidase (CCO) and water; (2) improved quantification of chromophore concentration when compared to two-wavelength systems by resolving complete spectral features; and (3) capability to obtain pathlength information and absolute concentration of chromophores via second differential spectral approaches.^51^

Hyperspectral and broadband setups require a “white” light source. Traditionally, this source is a fiber-coupled continuous wave halogen-based lamp. Developments in the telecommunications industry have enabled miniaturized LEDs with broadband NIR enhanced spectra (350–1000 nm) and low power consumption that can be surface mounted to flexible circuit boards. Supercontinuum laser sources can also provide a “white” coherent light source with fast switching, enabling both CW and TD hyperspectral/bNIRS techniques, albeit with added cost and complexity.^52^ Examples of supercontinuum-based hyperspectral systems have recently been described elsewhere.^53^^,^^54^

The detection arm of a hyperspectral system is often a single camera (either CCD or CMOS-based technology).^49^ In contrast, bNIRS devices are exclusively based on the utilization of a spectrometer that incorporates a light wavelength dispersion grating with a camera that most often has been a CCD-based camera.^50^ While spectrometers have traditionally been large devices, the emergence of micro-spectrometers with enhanced detection sensitivity and dark noise suppression has provided several orders of magnitude of improvements in SNR. The challenges and solutions for using micro-spectrometers in bNIRS have recently been discussed by Kaynezhad et al.^55^ Micro-spectrometers have allowed for less intrusive and more portable devices that can be integrated within a healthcare/clinical environment. Recent developments in digital photography for mobile phones (with miniaturized lenses and high pixel count miniaturized cameras) and advances in CMOS technology and electronic component miniaturization allow us to start imagining wearable, fiberless hyperspectral/bNIRS devices assembled on a lightweight helmet on the head with micro-spectrometers of cubic centimeters in size. The challenge that remains to be tackled is to make these instruments easy to operate, easy to interface with other measuring systems, low-cost, and safe with broad applicability, real-time visualization, simple analytics, and enhanced brain sensitivity.

### 2.5 Diffuse Correlation Spectroscopy (DCS)

Diffuse correlation spectroscopy (DCS) is a biophotonic technique that is complementary to NIRS. DCS was first employed in 2001 to study cerebral blood flow (CBF) in animal models^56^^–^^58^ and then human subjects.^59^ Briefly, DCS typically uses fluctuations of collected light intensity to measure motions of scatterers in the light path.^22^ Measurement of these fluctuations is often derived from the temporal autocorrelation functions of the diffuse light, but equivalent information is encoded in the spatial correlations of scattered speckle. In tissues, the moving scatterers are predominantly red blood cells. Thus, faster or slower variations of the temporal light fluctuations reflect greater or lesser blood flow in the tissue vasculature. Validation of the connection between the average flow within interrogated tissue vasculature and the measured DCS temporal correlation functions was carried out in a variety of pre-clinical and clinical studies (e.g., in brain, cancer, and muscle tissues), and via comparison to ASL-MRI, Xe-CT, laser and ultrasound Doppler, and contrast-agent bolus uptake dynamics.^60^^,^^61^ Moreover, the utility of the DCS-flow metric as a biomarker has grown due to translational studies. For example, the combination of oxygen saturation and blood flow can be used to derive quantitative information about cerebral metabolic rate of oxygen (${\mathrm{CMRO}}_{2}$, Sec. 2.6); the combination of blood flow and blood pressure information can be used to probe cerebral autoregulation (Sec. 6.4.1), and rapid DCS-flow and blood pressure measurements with acquisition rates exceeding the heart rate can provide information about intracranial pressure (ICP, Sec. 6.4.2).

Looking forward, the utility of DCS could benefit from more insight into the microscopic origins of the signals. Like NIRS, DCS signals are coarse-grain averages over tissue networks, i.e., microscopic information on smaller length scales is averaged. Dual micro-macro investigation of the same tissues/tissue-types could help clarify the information content in macroscopic DCS measurements. Further, studies that characterize both molecular metrics and diffuse optical markers will help increase the clinical value of DCS. Finally, further examination of the (effectively Brownian) origins of the correlation function decay behavior is warranted; the current picture that flow-dependent shear stresses in vessels induce transverse diffusion of red blood cells is a great start,^62^^,^^63^ but more work is needed.

In a different vein, detected photons pass through the scalp, skull, and cerebrospinal fluid, and therefore DCS measurements are susceptible to contamination from extracerebral hemodynamics. Indeed, brain sensitivity does not reach levels where extracerebral sensitivity is small until source-detector separations are ∼3  cm. At such separations, fewer photons are available for detection, resulting in low SNR. Recently, the field has experienced spectacular technical progress with variants of DCS based on collections of correlation functions derived from many speckles or many modes. These approaches can dramatically improve SNR. Additionally, novel temporal gating methods to separate photons that have traveled long distances from those that travel short distances hold potential to improve sensitivity to cerebral perfusion. Herein we highlight new developments and approaches with potential to facilitate future applications.

#### 2.5.1 High-density single photon avalanche diode (SPAD) array detection

Recent work has incorporated high-density SPAD arrays for parallelized DCS detection of over a thousand individual speckle modes at close to megahertz sampling rates.^64^^–^^66^ By treating each SPAD pixel within an array of N pixels as an independent decorrelation rate detector and averaging the result across all pixels, SPAD array-based parallelization can improve the SNR of DCS by a factor of the square root of N.^65^^–^^67^ SPAD arrays have recently enabled the detection of mm-scale perturbations buried up to 1 cm deep underneath tissue-like phantoms at up to 33 Hz, as well as monitoring of blood flow from above the prefrontal cortex at up to 30 ms temporal resolution to resolve ventricle contraction and repolarization within the pulse.^65^ Parallelized DCS systems have also been piloted in monitoring CBF in humans during tasks expected to elicit prefrontal cortex activation.^65^ Apart from directly averaging the DCS signal across all SPAD pixels within an array, the signal from individual SPAD pixels may also be digitally combined and processed in alternative configurations, for example, to form images and video of deep-tissue dynamics.^68^

Currently, one main limitation of SPAD array-based DCS detection is its relatively low per-SPAD temporal sampling rate. While a single SPAD can sample at over 100 MHz,^69^ SPAD arrays currently operate at approximately 1 MHz frame rates. This acquisition rate currently limits their application for detecting rapid speckle fluctuations arising from tissue depths necessary to sense the brain (sub-ns to μs). A second current limitation of SPAD arrays vs. single SPADs is a lower photon detection efficiency, which we anticipate will increase as integrated solid-state CMOS detector fabrication processes improve. In addition, demonstrations of DCS with SPAD arrays to date have utilized just slightly over one thousand pixels, compared with the millions of pixels currently available in a standard CMOS or CCD pixel array. Luckily, high-density SPAD arrays with up to one million pixels have recently emerged.^70^^,^^71^ In the future, we can likely expect the speed, sensitivity, and pixel counts of such novel integrated arrays to follow the general trends of improvement that are well-known within the semiconductor industry to benefit upcoming DCS platforms continually.

#### 2.5.2 DCS at 1064 nm

Traditionally, DCS has employed source illumination in the NIR between 785–852 nm. Recent work has investigated the use of longer source wavelengths for DCS. Between 1050 and 1100 nm, water has a local absorption minimum, offering an additional transmission window for deep tissue measurements, and tissue scattering is progressively reduced. Although this spectral window is less useful for NIRS because hemoglobin absorption is very low, DCS is not impacted as it relies on the dynamic scattering of light by red blood cells, which remains substantial in this wavelength range. The 1064 nm wavelength represents a favorable operating point due to the wide availability of laser sources, fiber amplifiers, and other optical components first developed for the communication industry.^72^

One of the benefits of 1064 nm operation is that the effective attenuation coefficient ($\mu_{\mathrm{eff}}(\lambda )\equiv \sqrt{3\mu_{a}(\lambda )\mu_{s}^{\prime }(\lambda )}$) is 10–20% lower than in the 650–850 nm range, offering improved light penetration and brain sensitivity. In addition, ANSI standard limits for safe skin exposure (ANSI Z136.1) allow 2.7–3.7 times more energy to be delivered per unit area than 785–852 nm. Further, because photons carry less energy at longer wavelengths, ∼40% more photons are expected per unit of energy at 1064 nm compared to 785 nm. Finally, the intensity autocorrelation decay is slower at 1064 nm due to the decreased scattering, resulting in an increased SNR. Altogether, these factors have been shown to result in 10–20 times more photons being detected at 1064 nm compared to 785-852 nm, corresponding to 10–20 times SNR gain for DCS measurements at 1064 nm. This SNR advantage can be traded for either increased temporal resolution or increased source-detector separation. The 1064 nm operation can offer more than 1 cm extension of the maximum source-detector separation at the same SNR when compared to 785–852 nm DCS measurements, making 3–3.5 cm separations practical and resulting in a 2–3 times increase in the ratio of cerebral to extracerebral blood flow sensitivity.

The main challenge for DCS at 1064 nm is the lack of suitable semiconductor detectors. Current commercially available silicon SPADs have very low efficiency, while indium gallium arsenide (InGaAs) SPADs have excessive after-pulsing. Thus, the full potential of DCS at 1064 nm has so far been demonstrated only using cryo-cooled superconducting nanowire single-photon counting detector (SNSPD) devices that have limitations due to cost, size, and noise.^73^ Novel gated InGaAs detector arrays are currently under development to be used with a pulsed laser source. These are expected to offer sufficient channels to implement cross-correlation detection to remove after-pulsing effects and will be able to power large functional DCS imaging probes.

#### 2.5.3 Time-domain DCS (TD-DCS)

The development of time-domain DCS (TD-DCS)^74^ has enabled the selection of photons with long path lengths by their time-of-flight to minimize the influence of extracerebral layers on the measured blood flow index. This approach can be applied even at short separations where more detected photons are available—the same principle behind the “null-separation” TD-NIRS approach.^75^ TD-DCS systems comprise a picosecond pulsed laser (∼300  ps FWHM pulses appear to offer the best balance between coherence and the ability to resolve deep traveling photons) and time-correlated or gated single-photon counting detectors that allow the selection of photons arriving in a specific time window (gate) after the laser pulse. The intensity autocorrelation is computed using only photons arriving “in the gate” over the same time intervals typically used for DCS (sub-ns to ms). TD-DCS has been piloted in measuring the response to physiological manipulations and preliminary assessment of functional brain responses.^69^^,^^76^^,^^77^ A key advantage of TD-DCS is the MHz laser repetition rates used, which allows for the implementation of dense optode arrays without crosstalk, as multiple illumination states can be employed without any loss of temporal resolution or SNR. Currently, the main limitation of TD-DCS is the lack of efficient detectors without a “diffusion tail” affecting their timing response, as well as the higher cost of components versus traditional CW-DCS systems.

In the near future, we expect TD-DCS to benefit from long-wavelength operation at 1064 nm and the development of new detector technologies. On the one hand, SNSPDs have recently emerged as the ultimate detector technology with very high photon detection efficiencies across broad wavelength ranges, nearly negligible dark counts, and no after-pulsing.^73^ However, as mentioned in the previous section, SNSPDs are expensive, large, and loud, though miniaturization efforts are ongoing. At the same time, SPAD cameras with thousand element and larger detector arrays are reaching higher performance levels in terms of detection efficiency and timing performance.^78^ These improvements will permit the practical implementation of TD-DCS imaging arrays for functional brain measurements across the whole head.

#### 2.5.4 Interferometric diffusing wave spectroscopy (iDWS)

Recently, interferometric optical methods have also been investigated for measuring diffuse light fluctuations arising from CBF dynamics.^79^ The basic idea is to split a portion of the source light into a reference path that recombines (or interferes) with the light that has reached the detector after diffusing through the tissue. In contrast to traditional DCS, which measures intensity fluctuations to determine the temporal intensity autocorrelation, interferometric DWS (iDWS) measures electric field fluctuations of the sample light that are in-phase with the reference light^79^ to determine the temporal field autocorrelation. In iDWS, the strong reference light field multiplies and amplifies the weak sample light field from the tissue, enabling users to replace the photon-counting detectors of DCS with noisier, inexpensive detectors. Because the sample signal is amplified, iDWS is relatively insensitive to ambient light. Array detectors in conjunction with multimode fiber collection can parallelize iDWS and improve light throughput and SNR.^79^ Interferometric techniques have been demonstrated with an APD,^80^ a fast-sampling linear array sensor (e.g., non-scientific line-scan CMOS camera^79^), and in the Fourier domain, with a two-dimensional sensor.^81^ Path length discrimination, akin to TD-NIRS and TD-DCS, can also be achieved in an interferometric setup by reducing the coherence length of the light source.^82^^,^^83^

The SNR of a single interferometric channel can exceed that of a standard DCS photon-counting channel^80^ if the reference light level is high enough. Therefore, with parallel detection, iDWS greatly improves SNR and reduces cost, accessing higher temporal resolution and allowing for measurements at larger source-detector separations.^76^ For instance, pulsatile iDWS BFI measurements at 3.5-4.0 cm source-detector separation were recently demonstrated with a 333 kHz line-scan CMOS camera, where ∼192 interferometric channels were achieved.^76^ Further improvements of the SNR-to-cost ratio will be possible with iDWS methods that utilize low frame rate two-dimensional sensors^84^^,^^85^ with many megapixels.^80^^,^^85^ Keeping in mind that the field autocorrelation decays at half the rate of the intensity autocorrelation, detectors that provide access to μs time scales are needed to maximize brain specificity. While the original iDWS approach^86^ required rapid sampling, the multi-exposure approach^86^ allows access to these time scales with short exposure times. Thus, we anticipate that the next five years will see increasing adoption of interferometric optical CBF sensing and mapping in human subjects.

#### 2.5.5 Speckle visibility spectroscopy (SVS)

Speckle visibility spectroscopy (SVS), also known as speckle contrast optical spectroscopy (SCOS)^87^ or diffuse speckle contrast analysis (DSCA),^88^ is based on the idea that a rapidly fluctuating speckle field imaged on a camera with a fixed integration time will appear washed-out and more uniform than a more slowly fluctuating speckle field.^89^ A sensitive determination of the speckle decorrelation can be made by characterizing the overall speckle statistics. SVS is categorically a DCS method, but it differs from most traditional DCS methods in that it measures speckles in space (ensemble) rather than in time (temporal) to arrive at an index of blood flow in the underlying tissue. The sensitivity of both strategies is unified by the number of independent observables (NIO) measured.^90^ In a traditional temporal DCS measurement architecture, a single speckle grain is measured over time with a sensitive single-pixel detector, and the NIO is the number of full decorrelation events measured.^61^ In speckle ensemble DCS measurements or SVS, speckle grains are measured in parallel on an area detector such as a camera, and the NIO is the number of speckle grains imaged on the sensor.^89^ The SVS approach allows higher NIO to be attained by leveraging high pixel count cameras.

**Fig. 1:**
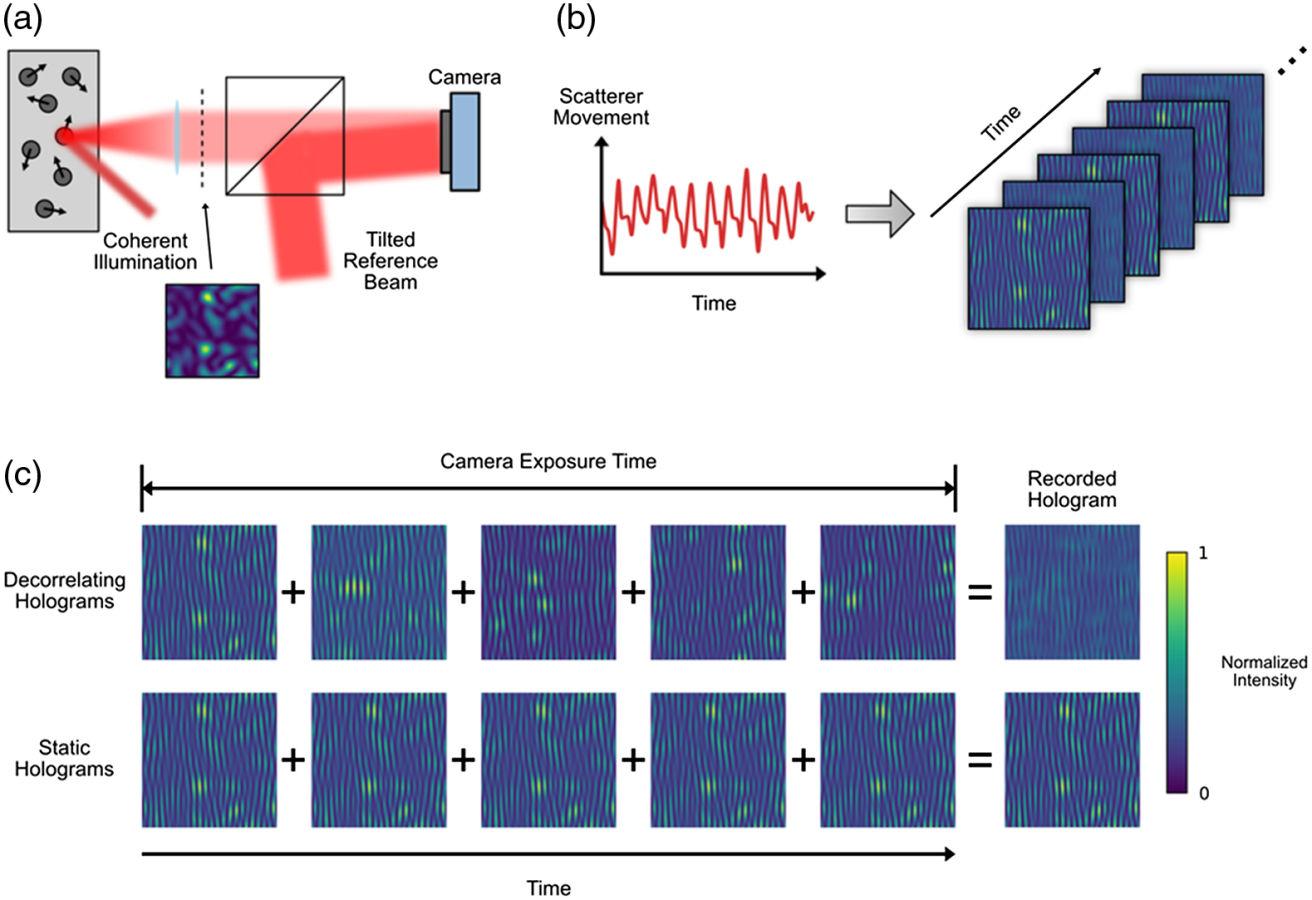
Measurement of scattering dynamics with interferometric speckle visibility spectroscopy (iSVS). (a) Coherent light illuminates a dynamic scattering media, generating a time-varying speckle field. The speckle field is combined with a tilted reference beam to form a hologram on the sensor. (b) The movement of scatterers (e.g., red blood cells) within the sample create captured holograms with normalized intensities proportional to the speed of the scatterer movement. (c) A depiction of how the recorded holograms are formed. If the medium is dynamic, the speckle pattern changes during the camera exposure time, leading to a reduced overall speckle contrast and maximum intensity in the recorded hologram. In contrast, if the scattering medium is static, the captured speckle pattern integrated over the exposure time is static, leading to a higher contrast captured hologram. (Figure modified from Xu et al.^90^)

SVS can be further improved by adding a reference beam to enable interferometric measurements—a method termed interferometric speckle visibility spectroscopy (iSVS).^84^ The iSVS scheme is illustrated in Fig. 1. A standard SVS setup is modified by interfering the captured speckle field from the sample with a tilted reference beam, forming a hologram on the sensor. The hologram can be analyzed using standard off-axis processing techniques to retrieve the sample field.^91^ The amplitude of the speckle pattern captured in the hologram is related to the medium’s decorrelation time, which is ultimately associated with the flow within the medium.

iSVS can perform shot-noise limited detection even in photon-starved situations, enabling the use of commercially available, large pixel count, high-speed cameras with higher noise levels than the sensors needed for SVS. The higher NIO can be leveraged to either improve the measurement’s SNR or to enable parallel measurements. One limitation of techniques based on spatial ensemble measurements is that if the underlying decorrelation process is associated with multiple decorrelation time constants, SVS and iSVS will not be able to separately quantify these constants. On the other hand, SVS and iSVS can reliably output an aggregate decorrelation time measurement, which is well suited for characterizing dynamic changes in the brain due to brain activity. iSVS also shares the limitation of other DCS type measurements in that the depth sensitivity relies on the distribution of the photon paths and thus degrades with penetration depth. However, the interferometric nature of the detection provides the ability to modify the coherence of the light source to improve the depth selectivity.

In the next five years, both practical and theoretical concerns of iSVS should be addressed to improve the performance and to enable it to be more easily used in laboratory and clinical settings. Practically, designing a stable and robust light collection system is critical to isolating the scattering dynamics from other environmental factors such as vibration. Other theoretical avenues for exploration include optimizing the source parameters (e.g., wavelength, coherence length) to maximize the sensitivity to the dynamics of interest and exploring the use of multi-exposure methods.

### 2.6 Hybrid NIRS and DCS Systems

**Fig. 2:**
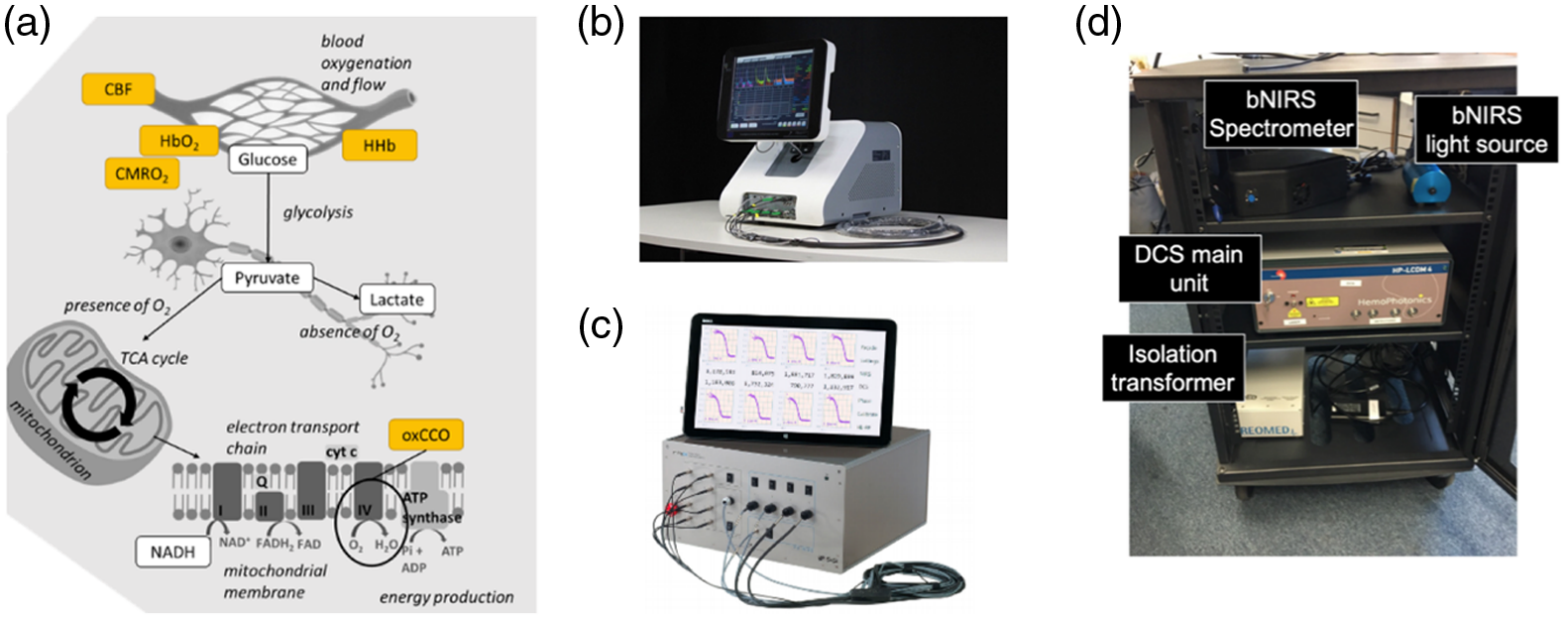
The power of combining diffuse optical techniques techniques (a) Yellow boxes indicate the physiological measurements that can be achieved non-invasively with diffuse optical techniques, demonstrating the capacity to interrogate from the vascular system down to the mitochondrial and the tricarboxylic acid (TCA) cycle, also called Krebs cycle, for adenosine triphosphate (ATP) energy production (CBF: cerebral blood flow; HbO2: oxygenated hemoglobin; HHb deoxygenated hemoglobin; ${\mathrm{CMRO}}_{2}$: cerebral metabolic rate of oxygen; oxCCO: oxidized cytochrome-c-oxidase). Figure modified from Bale et al.^94^ (b) The BabyLux^95^ system combines a two-wavelength time domain near-infrared spectroscopy (TD-NIRS) module with a dual-channel DCS module.^96^ It includes highly customizable software suitable for medical personnel with a self-guided software/hardware procedure. It has been developed within a European project funded by the European Commission (No. 620996) and is available as a custom system through HemoPhotonics S.L. (Spain). (c) The MetaOx system combines an eight wavelength, four-channel frequency domain NIRS (FD-NIRS) module with an eight-channel DCS module.^97^ It has been developed in collaboration with ISS, Inc. (Champaign, Illinois, USA) and the Massachusetts General Hospital (Boston, Massachusetts, USA) and is available as a research system through ISS, Inc. (d) The hybrid system comprising both broadband NIRS (bNIRS) and DCS recently developed at the University College London (London, UK).

Since its introduction in the mid-90s, DCS has been utilized alongside NIRS in many experiments^22^^,^^60^^,^^61^^,^^92^^,^^93^ and, lately, in commercial devices (Fig. 2). It is commonly combined with FD- or TD-NIRS, although some studies have also incorporated DCS with CW-NIRS. There are two key driving reasons for this combination.^61^ The first reason is purely physical: the correlation diffusion model used to analyze DCS data depends on $\mu_{a}$ and $\mu_{s}^{\prime }$. While the relative changes of CBF measured with DCS are largely independent of these parameters, the absolute assessment requires that they are estimated independently.^98^ The second reason is physiological: the combination of these two tools yields more information than the sum of their parts about the balance between delivery, availability, and extraction of oxygen in the interrogated tissue volume [Fig. 2(a)]. In other words, in addition to measurements of hemoglobin oxygenation, blood volume, and blood flow, ${\mathrm{CMRO}}_{2}$ extraction can be estimated, which is, in principle, a complete marker of whether the interrogated tissue can meet the demands of the brain function through the oxidative metabolism. The combination of NIRS and DCS can significantly transform brain monitoring when delivered in a highly portable package that can be employed across environments and populations.

The first hybrid NIRS/DCS demonstration on the adult human brain used an interleaved, time-sharing mode to estimate CBF, HbR, HbO, and ${\mathrm{CMRO}}_{2}$ near-simultaneously.^59^ To date, this approach continues to dominate the field of diffuse optics. More recently, optical filters have been introduced to enable simultaneous illumination of the tissues increasing the acquisition speed and throughput,^96^^,^^97^ while others have utilized multiple wavelengths and DCS detectors as a combination of CW-NIRS and DCS.^99^ Recent reports show simultaneous acquisition rates higher than 10 Hz for both FD- and TD-NIRS and DCS, which will open new avenues to study fast hemodynamic/metabolic signatures of cerebral function and health. The combination of bNIRS with DCS has also been demonstrated recently.^100^^–^^102^ New commercial devices are emerging through international consortia, e.g., VASCOVID, TinyBrains, and the NIH BRAIN Initiative, moving towards large-scale clinical trials of combined NIRS/DCS. The future of hybrid instrumentation is expected to move towards portable/wearable systems, and to systems with tens of channels leading towards high-density tomography. Moreover, with the maturation of technology, time-domain (Sec. 2.3), interferometric (Sec. 2.5.4), and speckle contrast-based (Sec. 2.5.5) approaches that can simultaneously provide quantitative information about optical properties and flow will become more prevalent. These new data types are expected to provide more efficient and portable hybrid instrumentation.

### 2.7 Instrument Comparison and Standardization

Standardized instrumentation performance assessment and dedicated phantoms are of great importance to achieve comparability and reliability in diffuse optical measurements. The diffuse optics community is particularly active in pursuing multi-laboratory initiatives for joint testing of instruments. Three protocols for performance assessment of diffuse optics instruments (BIP,^103^ MEDPHOT,^104^ and nEUROPt^105^) developed in European projects are state-of-the-art for instrumentation characterization in this field. BIP addresses the basic instrumental performance primarily of time-domain instrumentation on a hardware level. MEDPHOT evaluates the capability to measure $\mu_{a}$ and $\mu_{s}^{\prime }$ of homogeneous diffusive media. The nEUROPt protocol targets inhomogeneous conditions, specifically fNIRS imaging, by characterizing detection (contrast, contrast-to-noise ratio), localization (lateral resolution, depth sensitivity), and quantification (accuracy, linearity) of absorption changes in the brain. The recent BitMap exercise for cross-comparison of diffuse optics instruments applied these three protocols and shared phantom kits to compare 28 systems from 12 institutions in 7 countries.^106^^,^^107^

IEC/ISO international standards have been developed recently for (CW) functional NIRS equipment^108^ and cerebral tissue oximeters^109^ as medical electrical devices. These standards also include phantom-based performance tests, but they do not yet cover testing for brain selectivity in particular, which is extremely important for both types of equipment. Future directions of phantom-based performance assessment will be widespread commercial availability of the necessary phantom kits, a refined adaptation of test methods and phantoms to the clinical problems, related consensus publications, and raised awareness of these methodologies by device manufacturers and regulatory bodies.

The performance assessment of DCS instruments is still in its premature phase, with only individual or initial attempts to propose relevant metrics and to undertake performance comparisons, for instance, of laser sources for DCS.^110^ DCS is more recent and less widespread than NIRS and multi-laboratory initiatives are mostly needed to reach consensus on common metrics and key figures.

### 2.8 Instrument Commercialization

Both progress in instrumentation and the quick adoption of diffuse optics by researchers in several fields have accelerated the translation of diffuse optical techniques to commercially available systems. A detailed summary of the current technology landscape on NIRS and DCS products made available through companies can be found on an online database accompanying this publication.^111^ While numerous commercial NIRS products have enjoyed market adoption since the early 1990s for both research and clinical use, we focused this assessment on the systems presently marketed for purchase at the time of writing, with an emphasis on devices developed for brain monitoring. Conversely, given the landscape of DCS products is comparably nascent, we include any product from a commercial entity with publicized plans to commercialize for research or clinical settings.

Broadly speaking, commercially available NIRS systems are categorized as either bedside-portable (Ref. 111, Table S01) or wearable/wireless systems (Ref. 111, Table S02). Bedside-portable devices represent the earliest entrants to commercial translation. Wearable/wireless systems reflect the recent paradigm shift in the commercial market responding to consumer preferences for digital health technologies that are ergonomic, highly portable, and lend data-driven insights into consumer lifestyle (e.g., smartwatches). Of the products identified at the time of writing and reflected in Tables S01 and S02, wearable/wireless systems comprise the majority of commercial systems (63% vs. 37%). Unsurprisingly, the vast majority of bedside-portable and wearable systems utilize CW-NIRS approaches (76% and 88%, respectively). Of these, HD-DOT arrays comprise a relatively equal proportion of bedside-portable devices (53%) and wearable systems (51%). FD-NIRS and TD-NIRS modalities are a limited, but emerging segment of the technology landscape. Four bedside-portable FD-NIRS devices (16%) are developed by ISS, Inc. (USA), one bedside TD-NIRS device is exclusively marketed in Japan for clinical use (tNIRS-1, Hamamatsu, Japan) and two wearable TD-NIRS devices recently developed by startups within the last 2 to 3 years are available as research devices (NIRSbox by PIONIRS, Italy; Kernel Flow by Kernel, USA).

To date, there is only one commercially available DCS product (MetaOx by ISS Inc., USA; see Ref. 111, Table S03). While the commercialization of DCS-based neuromonitoring is in its infancy, an exciting era of technology transfer is emerging as two research groups have spun-out well-established, early-stage startup ventures intending to commercialize DCS for clinical use.

Over the next five years, we anticipate that commercialization of NIRS devices will continue its present expansion in developing and marketing digital health and lifestyle/fitness products for research and consumer use. DCS devices will likely experience a comparatively rapid inflection in demonstrating proof-of-concept and financial milestones, progressing toward the first regulatory approval of DCS for routine clinical use. For example, collectively over the last decade, startups and small business ventures developing DCS technologies have raised at least $3.2M USD in non-dilutive capital to directly support technology transfer, technology development, and clinical validation. Given the clinical potential, as well as the need for wearable systems for functional neuroscience applications, we envision growth in these technology portfolios and the number of patients benefiting from commercialized products will expand exponentially in the next decade.

## 3 Data Analysis and Algorithms

Advances in optical hardware coupled with novel experimental protocols have driven cutting-edge data analysis strategies that have resulted in more reliable information extracted from the optical signal. Consequently, several algorithms have emerged as potential solutions to pitfalls like motion artifacts and extracerebral contamination, and standard data acquisition and analysis procedures have become necessary. This section discusses the current state-of-the-art of fNIRS data analysis and key considerations for data collection and interpretation. Most of the methods presented below were initially developed for CW-NIRS, which has been the widest adopted technique by users to date. However, these methods can readily be adapted to other diffuse optical approaches. Cases in which methodologies have been developed for specific diffuse optical techniques are also highlighted in each section.

### 3.1 fNIRS Data Quality Assessment

Diffuse optical signals are strongly influenced by experimental settings (e.g., motion artifacts, poor optode-scalp coupling) due to their typically low SNR. These influences impact fNIRS reproducibility at both the inter-subject and intra-subject levels and may adversely affect subsequent data processing and interpretation if not adequately addressed. Thus, the first step in data processing is to assess the quality of the recordings. This section focuses on several methods commonly utilized to enable informed decisions as to whether to preserve or discard certain data and/or whether to apply specific algorithms for reducing undesirable noise.

#### 3.1.1 Cardiac-based assessment of signal quality

Typically, fNIRS investigators consider the presence of cardiac pulsation in raw amplitude signals as a reliable and readily ascertainable indicator of successfully measured tissue hemodynamics. This feature has been translated into quantitative measures such as the Scalp Coupling Index (SCI)^112^ and the Peak Spectral Power (PSP).^113^ These complementary metrics quantify the strength of cardiac pulsation of raw fNIRS signals in temporal and spectral domains, respectively. Critically, they can be computed on each short-timed segment (3–5 seconds) of an entire fNIRS recording to capture its inherently time-variant quality adequately. Notably, the combination of SCI and PSP allows for robust discrimination of clean signals from movement artifacts and/or noisy signals.^113^

Of practical note, such quality assessment can be performed and displayed for all fNIRS optical source-detector separations (i.e., channels) both in real-time during the headgear fitting to optimize the setup (e.g., PHOEBE)^113^ as well as *post hoc* to assess the data quality prior to other processing steps (e.g., QT-NIRS).^114^ In the latter case, data quality assessment and pruning of channels with little to no cardiac pulsation can be carried out automatically alongside data analysis by directly interfacing QT-NIRS with analytical tools such as Homer 3 or Brain AnalyzIR as part of the same graphical or programmatical pipeline. In addition, QT-NIRS can evaluate multiple recordings as a batch to generate a study-wide data quality report. At this time, extensive validation of these measures and technologies is being performed using both ground-truth datasets and real-world datasets encompassing a wide variety of experimental protocols and populations. It is expected that additional quality measures from existing and new literature will be incorporated into fNIRS data analysis in the near future.

#### 3.1.2 Motion artifact correction

Motion artifacts are one of the primary sources of noise in optical data, particularly in experiments involving infants or challenging clinical populations, as well as during tasks requiring participant movement (e.g., physical exercise or speech).^115^^,^^116^ Moreover, the development of wearable CW-NIRS devices has allowed monitoring participants while performing real-world activities,^13^ further increasing the chance that motion artifacts will affect the recorded signals. Several strategies and motion correction techniques have been proposed in the literature to reduce the occurrence and/or correct motion artifacts in fNIRS data.^117^^–^^122^ Recent comparison papers have demonstrated that a hybrid approach for motion artifact correction that combines spline and wavelet filtering seems to work best across several populations and tasks, especially when motion artifacts are highly contaminating the signal.^123^^,^^124^ This approach aims to first reduce baseline shifts, which are hard to correct with wavelet filtering, with the spline interpolation and then apply wavelet filtering to minimize all other types of motion artifacts. The hybrid method was applied to different infant^123^ and adult^124^ datasets acquired with different NIRS devices, tasks, and laboratories worldwide with improved performance compared to other motion correction techniques.

One of the greatest limitations of most motion correction techniques is the requirement for users to set some input parameters, which might be dataset dependent, subjective, and quite challenging for non-expert users. In the future, we can expect the development of novel automatic motion correction techniques that will not require user input. For example, machine learning approaches might be applied to large fNIRS datasets to try to train a network able to correct any type of motion artifact,^125^ or one might demonstrate that the input parameter of the wavelet technique might be automatically computed based on some known features of the experimental design (e.g., length of stimulus or SNR). Furthermore, integrating motion sensors in recently developed modular wearable fibreless NIRS devices will be straightforward, and novel motion correction approaches could be extended to exploit this additional information.^126^

#### 3.1.3 Spatial registration

Previous test-retest fNIRS studies have shown that different sessions conducted on the same subject within a short interval can yield highly variable results.^127^^–^^132^ This lack of reproducibility limits group-level analysis as well as within-subject comparisons across time in longitudinal studies. Although the high fNIRS variability is often attributed to low SNR, motion artifacts, and physiological noise,^127^^–^^132^ recent works have shown that the lack of spatial information plays an important role in the observed fNIRS variability, particularly in low-density probes.^132^^,^^133^ Variability in probe placement across subjects or sessions increases the fNIRS variance and reduces statistical power in data analysis. In some cases, marginal changes that are deemed statistically significant may merely reflect heterogeneity from underlying anatomy rather than brain functional changes.

**Fig. 3:**
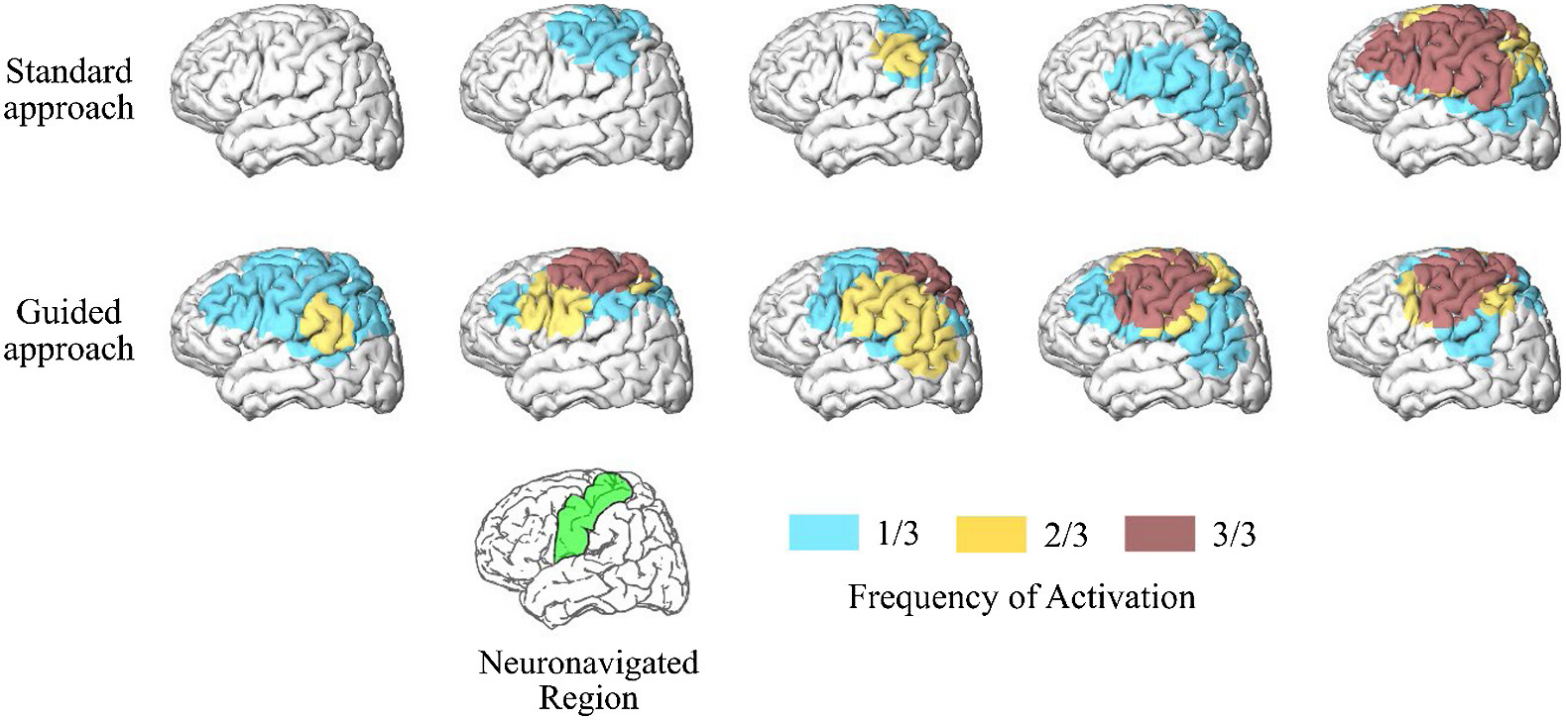
fNIRS reproducibility with a neuronavigation system. Frequency of activated brain regions during a motor task for the standard and guided approaches for probe positioning across five participants. The standard procedure used a tape to record head-size and find the optode location relative to the 10-20 system, while the guided approach used real-time neuronavigation software to place the optodes on the target region of interest (motor cortex, shown in green in the reference brain located at the bottom of the figure). Data were collected at three sessions on three different days for every subject. The frequency of activation represents reproducibility.

Methods employing probe registration have been previously proposed in the fNIRS literature to overcome this limitation.^134^^–^^137^ Typically, a digitizer and/or camera are used to acquire the position of the optodes, which can later be registered onto an anatomical MRI/CT image or a 3D model of the brain. Despite the effort to record this spatial information, there has been little development of data analysis methods to incorporate the probe location into the fNIRS analysis. One readily available solution recently presented is to use a forward model of light propagation to perform a region of interest (ROI) or voxel analysis of the fNIRS data rather than standard examination in the channel space.^138^ Another powerful alternative approach employs real-time neuronavigation software to guide the optode positioning.^132^^,^^133^^,^^139^ With real-time feedback, one can precisely position the optodes on the subject’s head and guarantee the NIRS optodes are on the target ROI before any measurement is taken, thus reducing variability due to spatial imprecisions. When applied to a longitudinal study, this approach doubled the intra-subject reproducibility and increased the fNIRS sensitivity to detect hemodynamic changes during a motor task (Fig. 3).^132^ As fNIRS advances towards routine functional brain imaging, stable longitudinal results at the subject level will become indispensable. Therefore, we envision that assisted probe positioning will be standard in longitudinal fNIRS protocols. Moreover, we envision this strategy could be extended to clinical settings to collect accurate optical data above a focal brain injury with a few sources and detectors, which will speed up experiment setup and data collection.

### 3.2 Extracerebral Contributions to the Optical Signal

The diffuse optical signal represents a mixture of different components originating from cerebral and extracerebral sources as well as neuronally and non-neuronally induced changes.^140^^–^^142^ Numerous approaches have been put forth to minimize or remove these components, which we highlight here.

#### 3.2.1 Systemic physiology augmented fNIRS

The primary source of the non-neuronal influence on fNIRS signals arises from systemic physiology, which can induce hemodynamic changes in both the extracerebral and cerebral tissue compartments. Thus, not all fNIRS-measured signal changes result from neurovascular coupling nor reflect brain activity. Changes in respiration, blood pressure, or autonomic nervous system activity can all induce changes in fNIRS signals that may mimic hemodynamic responses induced by neurovascular coupling.^140^^,^^141^ One way to optimally interpret fNIRS signals is by adding independent measurements of key systemic physiology parameters, including arterial oxygenation (${\mathrm{SpO}}_{2}$), respiration rate (RR), heart rate (HR), end-tidal carbon dioxide (${P_{\mathrm{ET}}\mathrm{CO}}_{2}$), continuous blood pressure (mean arterial pressure, MAP, and pulse pressure, PP), and skin conductance (SC). Measuring systemic physiology in combination with NIRS has been termed “systemic physiology augmented fNIRS” (SPA-fNIRS).^143^ SPA-fNIRS has been applied in several studies to investigate the physiological reaction of subjects during fNIRS acquisitions, and significant changes in systemic physiology were found even during easy tasks that are not strenuous, such as exposure to colored light.^144^^–^^146^ SPA-fNIRS can also be used in a hyperscanning setting; the first SPA-fNIRS hyperscanning study was recently published.^147^

In general, SPA-fNIRS is an excellent approach to enable a deeper understanding of the fNIRS signals and prevent misinterpretation. It allows one to gain insight into what causes the changes in fNIRS signals. In addition, SPA-fNIRS can generate a novel framework to investigate the complex inter-relationship between brain activity-related cerebral hemodynamics and systemic physiological activity. Current development of SPA-fNIRS concerns the optimization of the multimodal measurement setup as well as signal-processing and data analysis frameworks tailored to exploit the full potential of SPA-fNIRS. When possible, SPA-fNIRS should be increasingly used in future fNIRS studies since there is growing evidence that multi-distance approaches contain most but not all systemic information.^148^

#### 3.2.2 Multi-distance fNIRS

In fNIRS, various blind signal separation methods, such as independent component analysis (ICA), principal component analysis (PCA), or empirical mode decomposition, have been used to decompose the measured signal at a long separation into its components and to isolate the component that arises from the brain. (Note: Blind signal separation methods refer to approaches that attempt to isolate a signal when the source of the signal comes from a set mixed signals in which the mixing methodology is unknown.) However, these methods have the risk of overcorrecting the signal by removing the frequency band of interest.^149^ Using the average of all long source-detector separation measurements as a regressor representing the global systemic physiology also runs the risk of eliminating the evoked brain response, especially for sensor designs that cover only local regions of interest.

Short source-detector separation channels that measure only scalp hemodynamics are a robust solution for temporally filtering the confounding components in the fNIRS signals.^150^^–^^152^ With this approach, it is advisable to place short-separation detectors homogenously across the regions covered by the probe as the pial vasculature across the brain surface and the scalp vasculature are spatially inhomogeneous.^153^ A typical method of using the short-separation channel signal for the removal of confounding components in the fNIRS signal is to add the signal as a regressor in a General Linear Model (GLM) framework.^154^ This approach allows for a robust estimation of the brain response while regressing out the confounding signals. When enhanced with temporally embedded canonical correlation analysis, the method has been shown to further improve the estimation of the brain response by taking care of the time delays between different physiological signals.^155^

High-density multi-distance measurements are another approach that naturally allow for spatially filtering the hemodynamic changes in superficial layers through image reconstruction.^156^ Methods that incorporate short-separation regression directly into the image reconstruction scheme can further improve the estimation of the brain response.

While all these approaches are suitable for offline data analysis, regressing out the physiological confounds and extracting the brain response from fNIRS data in real-time requires more advanced methods. In this context, the Kalman filter allows a real-time estimation of hemodynamic changes for such data with dynamic statistical properties.^157^^–^^159^ As mobile NIRS systems will facilitate studies in the real world and in real-time, the fNIRS signal will become more prone to motion-induced artifacts and systemic interference. Novel data analysis approaches can benefit from multimodal regressors for systemic confounds and motion artifacts dynamically adapted in a Kalman filtering scheme.^160^

#### 3.2.3 Dual-slope method for enhanced depth sensitivity

Another approach to enhance depth sensitivity with multi-distance measurements utilizes two long channels (e.g., 2.5 and 3.5 cm) to generate data that have comparable contributions from superficial tissue and different contributions from cerebral tissue so that their combination can cancel out (or strongly suppress) extracerebral tissue contributions.^161^ This approach yields a slope of the optical signal versus source-detector distance, which is commonly accomplished in the field using a single source and multiple detectors (or a single detector and multiple sources).^162^^,^^163^ Because this approach uses a single element (source or detector), it is referred to as a single-slope method. An extension of the single-slope method was proposed in the late 1990s to achieve more robust measurements of the tissue optical properties with FD-NIRS and a special arrangement of two sources and two detectors (self-calibrating approach).^164^ Its main advantage is the insensitivity to probe-tissue coupling and instrumental drifts, thus allowing measurements without the need for any preliminary calibration. The special arrangement of two sources and two detectors for this self-calibrating approach has been used for cerebral oximetry with FD-NIRS^165^ and CW-NIRS^166^ and has been implemented in commercial cerebral oximeters based on CW-NIRS.^167^^,^^168^

Further characterization and development of the self-calibrating approach for individual intensity and phase slopes measured with FD-NIRS led to a so-called dual-slope method.^169^ The basic idea is to measure two paired single slopes (using two sources and two detectors), one where a given optode (say, a detector) collects data at the shorter distance and another where the same optode collects data at the longer distance. The dual-slope is the average of these paired slopes. This average cancels out or strongly suppresses contributions from source power, detector sensitivity, probe-tissue coupling, attenuation, delays in optical fibers, or any other instrument feature. In terms of probed tissue volume, dual-slope data were found to feature a greater relative sensitivity of deep vs. superficial tissue than single-slope and single-distance data, and the dual-slope phase showed a deeper region of sensitivity compared to dual-slope intensity.^170^^,^^171^ The value of a technique that is largely insensitive to instrumental drifts, probe-tissue coupling, and motion artifacts, and that is preferentially sensitive to deeper tissue in a spatially confined volume is highly attractive and particularly important in non-invasive optical measurements of the brain. The dual-slope method was used with CW-NIRS and FD-NIRS to measure cerebral hemodynamics,^170^^,^^172^ perform tissue imaging,^173^^,^^174^ and generate absolute broadband absorption spectra of turbid media and biological tissue.^175^^,^^176^

The current limitations of the method are related to signal-to-noise, especially in the case of phase measurements in FD-NIRS, and to the potential impact of lateral heterogeneity of tissue on the accuracy of the measured cerebral absorption changes. Nevertheless, the robustness and the preferential sensitivity to deeper tissue featured by the dual-slope method, especially with phase measurements in FD-NIRS, render it a valuable diffuse optical technique for functional brain imaging and the assessment of cerebral hemodynamics. Future developments include the characterization of the spatial region of sensitivity of dual-slope data in the presence of anatomical heterogeneity, the refinement of source-detector arrangements for imaging applications and optimal signal-to-noise conditions, and the selection of most effective modulation frequencies for specific applications.

#### 3.2.4 Depth selectivity with time-domain methods

The influence of extra-cortical contributions can be accounted for more easily using time-resolved methods,^177^ where the time-of-flight of photons can be related to the light penetration depth. In classical TD-NIRS, this can be done by recording the DTOF of photons using the time-correlated single-photon counting technique.^178^ Several data analysis approaches were proposed to estimate changes in the absorption coefficient of the medium at different depths in the tissue. These techniques are based on the parametrization of the measured DTOF: analysis of time windows, statistical moments, or Mellin-Laplace transform parameters.^43^^,^^179^^,^^180^ Combining time-domain with distance-resolved NIRS or multi-wavelength measurements improves the method’s depth-selectivity,^181^^,^^182^ including approaches based on two sources and two detectors that are arranged as described in the previous section for dual-slope measurements.^183^ An approach based on the null-distance technique was proposed in which depth discrimination is based on the direct measurement of time spent by photons in the tissue when the inter-optode distance is very short.^184^ Similar to TD-NIRS, one can also design an approach to estimate tissue perfusion at different depths using TD-DCS by analyzing the autocorrelation function with photons that arrived at a selected time-of-flight.^74^

These approaches have been validated in multiple *in vivo* experiments using neurophysiological tests wherein the cortical responses were selectively obtained and differentiated from extracerebral contamination.^54^^,^^185^^–^^187^ Potential utilization of the time-domain technique was also tested in several clinical applications.^188^ Application of modular, highly integrated multichannel systems providing measurements at multiple source-detector pairs will improve depth resolution and reduce the influence of lateral heterogeneity of extracerebral tissue.

#### 3.2.5 Multi-distance DCS

**Fig. 4:**
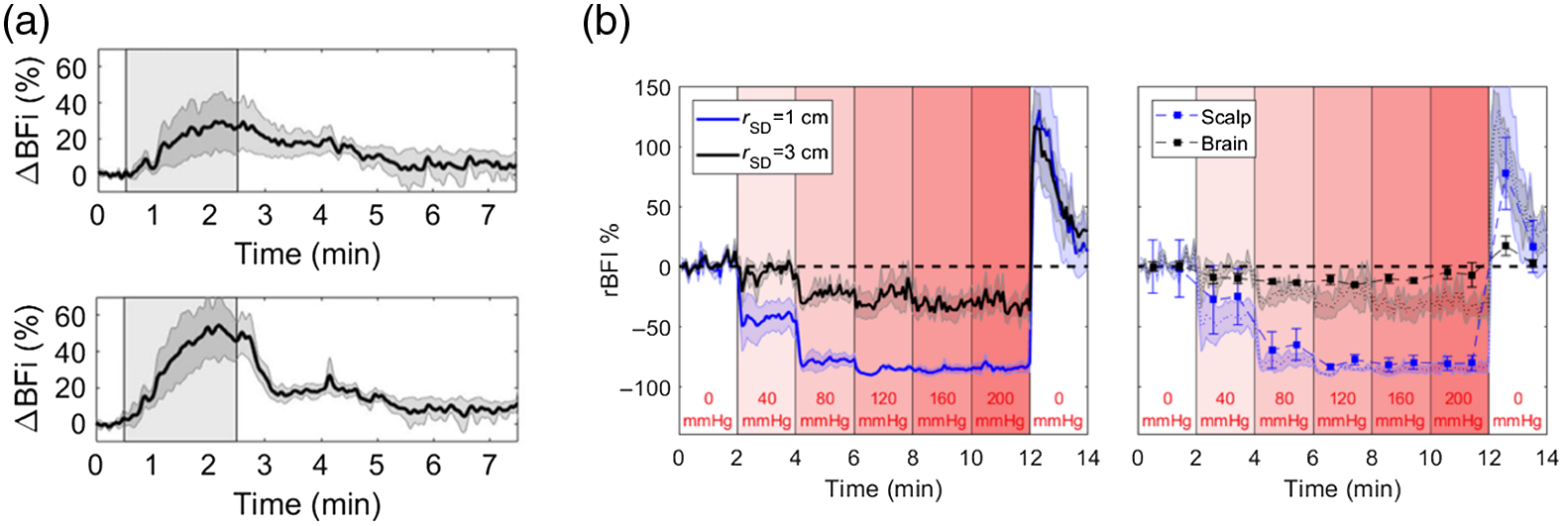
(a) Average increase in the blood flow index (ΔBFi) during a 2-min period of hypercapnia as indicated by the shaded region (N = 9). Time courses are presented for source-detector separations of 1 (top) and 2.7 cm (bottom). Shading surrounding each line represented the standard error of the mean. (b) Left: relative changes in BFI (rBFI) in response to increases in tourniquet pressure recorded at source-detector separations ($r_{\mathrm{SD}}$) of 1 and 3 cm. Shading around each line represents the standard error (N = 5). Right: rBFI for brain and scalp derived from the three-layer model. Error bars represent the standard error of the mean. For reference, the shading represents the individual time courses recorded at $r_{\mathrm{SD}}=1$ and 3 cm.

Compared to NIRS, the higher blood flow in the brain compared to the scalp gives DCS an inherent advantage in terms of depth sensitivity. Collecting DCS data at different source-detector separations provides the most straightforward method of monitoring flow changes in the scalp and brain. An example of this multi-distance (MD) DCS approach is seen in Fig. 4(a), which shows blood flow responses recorded at source-detector separations of 1 and 2.7 cm during a hypercapnia challenge. Notice how the response recorded at 2.7 cm was larger and had a more rapid decline after hypercapnia, indicative of the expected fast response of the cerebral vasculature to changes in arterial carbon dioxide tension.^189^

One approach to separate scalp and cerebral blood flow is to analyze MD DCS data using a three-layered solution to the diffusion approximation representing scalp, skull, and brain.^190^ The model requires independent estimates of the optical properties for the different layers and the thicknesses of the scalp and skull. The fitting parameters are blood flow indices for scalp and brain, assuming negligible flow in the skull layer. Despite the added complexity of the multi-layer model relative to the standard homogenous solution, Fig. 4(b) shows the benefits of separating the contributions from scalp and brain. In this example, DCS data were acquired at separations of 1 and 3 cm while inflating a cuff wrapped around the head to impede scalp blood flow. The model was able to predict negligible flow changes in the brain, as expected, despite large changes in the scalp, particularly at the end of the experiment when the cuff was released.^191^ A limitation with this modeling approach is the necessity to have independent measurements of layer thicknesses.^192^ In clinical applications, this information is often available from clinical MR or CT scans. Alternatively, it may be possible to use ultrasound to obtain these measurements,^172^ or to fit for this information by incorporating a simple pressure modulation challenge (designed to solely influence scalp perfusion) and constraining the fitting parameters accordingly.^193^

Other approaches to separate scalp and cerebral blood flow using MD-DCS include a modified Beer-Lambert law for flow or regressing out the signal from a short source detector separation using a general linear model (as in Sec. 3.2.2).^194^^–^^196^ While both of these approaches avoid several of the limitations of the three-layer analytical model and show promise as a means to isolate cerebral blood from extracerebral signals, work is needed to validate these approaches against other “gold standard” perfusion modalities before they become more widely utilized.

### 3.3 Tomographic Reconstruction of the Optical Signal

The use of overlapping data, primarily via high-density measurements, allows 3D spatial reconstruction of the underlying optical properties through diffuse optical tomography (DOT). Briefly, the principle of DOT is based on “back projecting” the measured data using a model-based optimization algorithm, often derived through computational models.^197^ Compared to conventional spectroscopy, the general benefits of DOT include enabling 3D spatial mapping of the measured functional data, allowing better localization, and inherently providing better quantitative accuracy of the recovered contrast. Although the NIR light interaction with tissue is non-linear, it is possible to recover 3D functional images (difference maps, similar to fNIRS) via assumptions regarding initial ground truth, both in terms of geometry and optical properties, but the use of atlas-based models has shown better accuracy.^3^^,^^198^

DOT systems employing time-resolved technologies are emerging, promising an unparalleled set of information regarding the imaged tissue.^199^ The detected time-of-travel of photons provides information to account for tissue absorption and scattering. Their intelligent utilization for spectroscopy and imaging has shown improvement in contrast and resolution and depth-dependent imaging.^43^

Currently, the main limitations of DOT are accessible data from multi-channel systems and efficient computational tools to allow image recovery in real-time. As systems develop that provide additional datasets, such as multiple-wavelengths, phase, and time-of-flight, both imaging and parameter recovery become more challenging due to the multi-parameter optimization required to derive many sets of unknown parameters from limited measurements.^200^ To allow quantitative DOT, in terms of absolute parameter recovery, the problem will become additionally challenging, as data calibration and accurate knowledge of the system response function becomes crucial, and their incorporation in any optimization and parameter recovery algorithm will be essential. Nonetheless, as we explore novel approaches in Machine Learning, we can expect that DOT will soon become the norm by providing quantitative functional data.^201^

### 3.4 Monte Carlo Methods for Diffuse Optics

The Monte Carlo (MC) method has been frequently used for modeling photon-tissue interactions in the brain.^202^^,^^203^ Because diffusion-based models^204^^,^^205^ can be inaccurate in the presence of cerebrospinal fluid (CSF),^206^ MC simulations provide a more accurate alternative to analytical models in the brain. Over the past decade, the research community has primarily focused on addressing two limitations of MC: low computational efficiency and the inability to model complex anatomical shapes. Many new MC publications embrace massively parallel computing architectures,^207^^–^^211^ such as graphics processing units (GPUs) or field-programmable gate array (FPGA), resulting in a dramatic shortening of typical simulation times from several hours on a single-core central processing unit (CPU) to only a few minutes or even seconds. Accurately modeling complex tissue boundaries is especially important for brain imaging.^206^ To this end, MC methods have made significant progress in recent years, permitting simulations in simple layered domains^202^ and complex heterogeneous tissues represented by 3D voxels,^208^^,^^212^ triangular surfaces, and tetrahedral meshes.^209^^,^^211^^,^^213^^–^^215^ Combined with advances in high-quality brain mesh generation,^206^ mesh-based MC^214^ shows particularly strong promise in modeling accuracy for brain applications. Despite these improvements, higher computational speeds remain among the highest demands in MC users’ wish lists. This need is further amplified as the community increasingly adopts high-density optical sensors and more sophisticated paradigms. Looking for new revenues to further accelerate MC computation, including image denoising^216^ and hybrid models,^217^ has been an active pursuit among MC developers. Also, to avoid slow computation, the bulk of the established optical brain imaging data analysis software pipelines has primarily focused on simplified head models and topological data analysis, even though more advanced modeling tools exist. As MC and brain modeling tools^206^ become increasingly efficient and accessible,^218^ we anticipate that many of these pipelines will incorporate 3D modeling^219^ and data analysis as part of their routine. This approach will provide users with increased accuracy and better integration with other resources made available by multimodal neuroimaging studies, including those with structural and functional MRI.

### 3.5 Brain Connectivity with NIRS

While traditional fNIRS uses a task-based approach to map function, there are also ways of using spontaneous, low-frequency (∼0.01−0.1  Hz) dynamics of the fNIRS signal at rest to map functions, connections, and networks. The ability to extract meaningful information about brain function at rest is particularly appealing in populations that cannot adhere to a task, such as infants^7^^,^^220^ or unconscious patients.^27^ In most cases, the main interest of these approaches is to unveil functional connectivity patterns through connectivity maps or networks,^4^^,^^6^^,^^148^^,^^221^ although it is also possible to estimate causal relationships across brain regions with effective connectivity patterns.^222^^–^^224^ These patterns can be quantified in both the time^6^^,^^225^ and frequency domain,^226^^,^^227^ and the recent release of connectivity-specific analysis toolboxes has made quantification of these patterns more accessible to a wide range of users.^228^^,^^229^ Moreover, recent work suggests that resting-state connectivity networks assessed with NIRS are repeatable at the single-subject level after accounting for extracerebral and systemic contributions, which may open doors for robust longitudinal studies.^148^

Moving forward, the ability to extract and quantify network properties will deserve special attention. The most common approach to quantifying connectivity, which uses data from one location as a “seed,” does not provide an integrated view of the brain or account for temporal variations in connectivity patterns. Alternatively, independent component analysis provides advantages in that it uses the whole dataset; however, it requires subjective determination of which independent component is associated with a given network. Graph theory offers an integrated view of the brain regions by quantifying the network’s topological properties based on the similarity matrix.^230^^–^^233^ The topological parameters calculated from the graph can be suitable markers for unveiling brain features related to functional communication.^234^^,^^235^ In addition, there is evidence that brain function and brain disorders affect the topological properties even at rest,^236^^,^^237^ despite challenges to compare differences between healthy and patient populations appropriately.^238^ While many approaches exist to quantify connectivity, future work will be needed to exploit the strengths of each approach to derive a robust, reproducible, and informative method to quantify these connections.

#### 3.5.1 Systemic low-frequency oscillations

Although functional connectivity maps derived from low-frequency oscillations (LFOs) are often attributed to neural activity, at least part of these oscillations appears to have a systemic contribution. The systemic low-frequency oscillations (sLFOs: 0.01∼0.1  Hz) can be a confounding factor for interpreting functional connectivity maps. On the other hand, sLFOs measured with NIRS can carry useful information on their own and may be biomarkers of disease severity in various pathological conditions that alter circulation (e.g., stroke).^239^

**Fig. 5:**
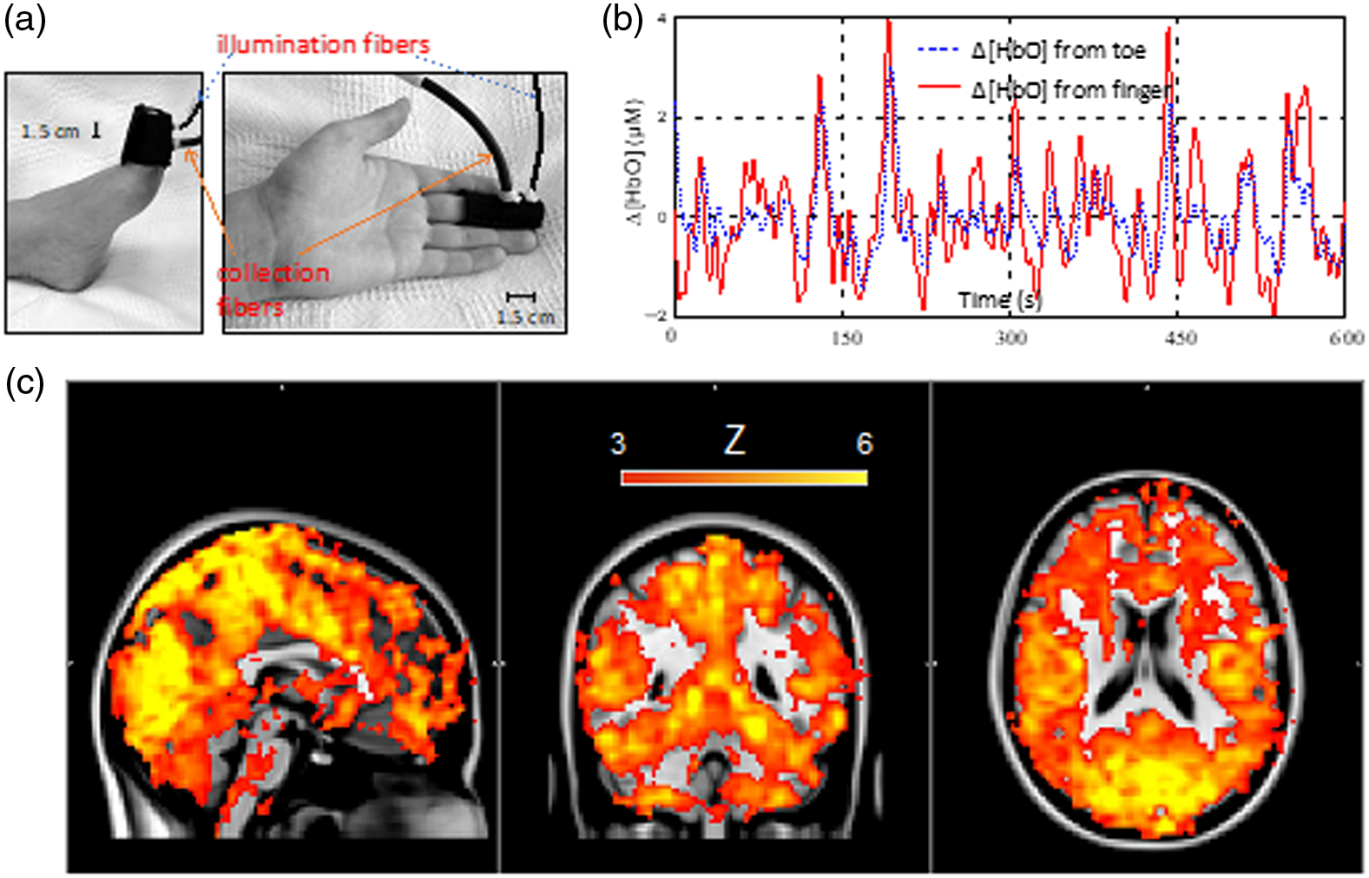
Concurrent peripheral NIRS measurements and resting-state fMRI. (a) NIRS sensors on the finger and toes. (b) sLFOs of changes in oxyhemoglobin concentration (Δ[HbO]) measured from the toe (blue) and finger (red). These sLFOs are highly correlated with a delay about 3 s. c) Map of voxels wherein the BOLD fMRI signal was highly correlated with sLFOs of Δ[HbO] measured in the finger at any time lag. Figure modified from Tong et al.^245^

The origin of sLFOs, which can be seen in both fNIRS and fMRI data, is still unclear. Several studies have explored the underlying mechanisms.^240^^–^^243^ In one of the early concurrent fNIRS/fMRI studies of resting state, LFOs in oxyhemoglobin concentration measured by NIRS in the prefrontal region were cross-correlated with the fMRI signal from each voxel. High correlations between fNIRS bandpass filtered in the LFO range and the fMRI signal were found in many brain voxels with various time delays (up to few seconds), and the dynamic patterns in the time delays of LFOs mimicked the patterns of blood flow distribution throughout the brain.^244^ The magnitude of the time delays of these correlations along with their spatio-temporal distribution indicates that the LFOs are likely associated with the global (i.e., systemic) blood flow and can propagate through the vasculature. For further validation, in another concurrent fNIRS/fMRI study, the NIRS sensors were positioned to monitor peripheral circulation (e.g., fingertips and toes). Again, high correlations were found between the NIRS sLFOs from the periphery and many voxels within the brain measured with fMRI. Moreover, the spatiotemporal patterns observed were similar to those found when using NIRS LFOs from the prefrontal cortex as a regressor (Fig. 5). Interestingly, the sLFOs located at the left and right fingertips were similar, while the sLFOs found at the toe were delayed approximately 3 s compared to that from the finger. These studies supported that sLFOs are global physiological oscillations associated with blood circulation. As a result, sLFOs can be used as biomarkers to assess circulatory patterns in the brain and even the whole body.^245^^,^^246^ Currently, the main limitation of this approach is the accurate identification of sLFOs because many physiological parameters (e.g., blood pressure) can contribute to sLFOs. In the future, we can expect that sLFOs will be used extensively in (1) identifying deficits in systemic circulation, and (2) denoising the fNIRS signal to expose the neuronal components.

### 3.6 Group-Level Analysis in fNIRS

Group-level analysis aims to make population-based inferences in the presence of different sources of variance, most prominently, within experimental unit variance and between unit variance. Capitalizing on solid theoretical grounds, regression analysis has traditionally dominated fNIRS group-level analysis using one- or two-level models (e.g., weighted linear regression and mixed-effects models).^247^^–^^250^ Traditionally, two-level models account for both within subject variance (first level) and between-subject variance (second level). A common variant of the two-level model is to count the frequency of a particular response (or any other feature) observed across volunteers in the first level (i.e., within-subject), which is essentially a change of variables to the traditional approach.^132^^,^^251^^–^^253^ This approach is mainly valuable for summarizing group responses when the inter-subject variability is high, such as in infants.^251^^–^^253^

The field is now exploring naturalistic settings that inherently introduce many confounding factors often not present in lab-controlled environments (e.g., the presence of distractors and additional extra-experimental stimuli). The demand for models dealing with large numbers of confounders propitiates the emergence of alternatives to classical regression, where modeling of the confounders is implicit. These include deep neural network models,^254^ which are favored due to the increase in available computational power, entropy-based modeling of variance with roots in Shannon’s information theory,^255^ or models coming from topological basis.^256^ The growing size of fNIRS datasets requires that group-level analysis methods scale well computationally. For such scalability demand, the traditional two-level approach is naturally well equipped. From mixing explicit-variance hierarchical and implicit-variance models, we can speculate that hybrid so-called summary models, where aggregated statistics are incorporated directly into the fitting algorithm,^257^ will eventually make their way into fNIRS analysis soon. In the meantime, analysis of small datasets can be performed using group-level analysis with reasonable power^258^ by addressing the effect of non-independence.^259^ Such theoretical advances have allowed us to revisit many early studies of smaller cohorts and will permit future exploratory studies where large cohorts are not viable (e.g., the study of rare diseases or of populations that are naturally constrained such as astronauts). In a different vein and breaking with Cantorian mathematics, fuzzy models such as clusterwise regression^260^ can take uncertainty into account and have capabilities for extended group regression. Fuzzy models have been recently used in fNIRS to study effective connectivity,^222^ and the translation of fuzzy models to fNIRS group-level analysis will probably be a matter of time.

### 3.7 Data Sharing and Data Standardization

The increasing utility of fNIRS has resulted in the technique being applied across an expanding range of research fields and applications.^261^ Consequently, the complexity of experimental paradigms and the amount of data acquired are rapidly increasing. To counterbalance this acceleration, tools and standards are required to facilitate reproducible neuroimaging research and to support researchers in conducting and reporting increasingly nuanced experimental analyses. To meet this demand, a variety of general-purpose analysis platforms and application-specific software tools^114^^,^^262^ have been developed. A summary of the existing tools is available in Table 1.

**Table 1:** Various software packages exist for analyzing fNIRS data, each with its own strengths and focus. The recent development of common file formats enables users to interchange between software and exploit the strengths of each package. Packages supporting the SNIRF file format and BIDS data structure at the time of publication are indicated in the table below.

Name	Language	Web Host	SNIRF Support	BIDS Support
*Data Analysis Packages*				
Brain-AnalyzIR^250^	MATLAB	https://www.nitrc.org/projects/AnalyzIR	Y	Y
FieldTrip^263^	MATLAB	https://www.fieldtriptoolbox.org	Y	Y
Homer^264^	MATLAB	https://github.com/BUNPC/Homer3	Y	Y
MNE-NIRS^265^	Python	https://github.com/mne-tools/mne-nirs	Y	Y
NeuroDOT^266^	MATLAB	https://github.com/wustl-orl/NeuroDOT	Y	Y
NeuroDOT^267^	Python	https://github.com/wustl-orl/NeuroDOT_Py	Y	Y
NIRFAST^197^^,^^268^	MATLAB	http://www.nirfast.org	n/a	n/a
NirsLAB^269^	Standalone	https://www.nitrc.org/frs/?group_id=651	n/a	n/a
NIRS Toolbox	Python	https://bitbucket.org/huppertt/nirs-toolbox/wiki/Home	n/a	n/a
NIRSTORM^270^	MATLAB	https://github.com/Nirstorm/nirstorm	Y	Y
PHOEBE	MATLAB	https://github.com/lpollonini/phoebe	n/a	n/a
QT-NIRS	MATLAB	https://github.com/lpollonini/qt-nirs	Y	Y
*Photon Migration Tools*				
AtlasViewer^135^	MATLAB	https://github.com/BUNPC/AtlasViewer	n/a	n/a
MCML^202^	Standalone	https://omlc.org/software/mc/	n/a	n/a
MCX^208^	Standalone/MATLAB	http://mcx.space/	n/a	n/a
MMC	Standalone/MATLAB	http://mcx.space/#mmc	n/a	n/a
MOCARTS^271^	Standalone/Java	https://github.com/javierherreravega/MOCARTS	n/a	n/a
NIRFASTer	MatLab	https://github.com/nirfaster/NIRFASTer	n/a	n/a
Toast++^272^	C++	http://web4.cs.ucl.ac.uk/research/vis/toast/	n/a	n/a

Standardized data storage formats are required to enable researchers to interchangeably utilize the strengths of each software and promote the sharing and reuse of data. These issues are not unique to the fNIRS community, and the path forward should be informed by experiences in other research-related fields.^273^ Recently, several community efforts have developed standards for the efficient sharing of fNIRS data. The Shared Near-Infrared Spectroscopy Format (SNIRF) provides a specification for storing fNIRS measurements. Software tools supporting SNIRF at the time of writing are indicated in Table 1, and some NIRS vendors already offer native support. To complement the SNIRF format, the Brain Imaging Data Structure (BIDS) formalizes metadata storage and specifies the organization of study files.^274^ Software is provided for validating and converting fNIRS measurements to BIDS format.^275^^,^^276^ Alternative formats, such as the NeuroJSON project^277^ and fNIRS-specific metadata format for LSL, are also in development. These initiatives will undoubtedly facilitate efficient sharing of fNIRS data and allow for storage and querying in structured online neuroimaging-specific storage services, such as OpenNeuro.^278^ Looking forward, software packages need to reflect recent best-practices guidance for analysis and reporting.^279^ In addition, consistent ontology^280^^–^^282^ and standardized software output^283^ will improve efficacy and reproducibility within the field.

## 4 Functional Applications in Neurodevelopment and Cognition

With the technological advances presented in Secs. 2 and 3 come exciting, novel opportunities to study the brain in subject cohorts and real-world environments wherein traditional neuroimaging approaches like MRI suffer significant drawbacks. These avenues include improved understanding of the developing human brain as well as for numerous applications in cognitive neuroscience. In this section we touch upon several of these emerging areas.

### 4.1 fNIRS in Neonates and Infants

Since the earliest days of near-infrared spectroscopy, the developing brain has been a key focus of research endeavors for three primary reasons.^284^^–^^286^ First, (and most pragmatic) the size and nature of children’s heads favor optical measurements: the small cranium, thinner scalp and skull layers, and in the case of babies, typically sparse (or absent) hair, ensure that the sensitivity of NIRS measurements to the developing brain is well beyond that of the adult.^44^ Second, both the vulnerable child in the clinical environment and the awake, active child make extremely challenging neuroimaging subjects, and more traditional methodologies such as MRI and fMRI (while not precluded) are very difficult to implement.^287^ Third, our brains experience greater structural and functional changes – and are more vulnerable to injury—in the first few weeks and months than at any other life stage.

Broadly speaking, the applications of neurophotonics in newborn and infant populations fall into two categories: those that seek to develop cot-side cerebral monitoring approaches to inform clinical management; and those that seek to use fNIRS and related approaches to advance our understanding of cognitive neurodevelopment. Both fields have made substantial technological steps forward in recent years. While the clinical advancements will be discussed in Sec. 5, in the context of functional neuroimaging, the newborn and infant populations are just now beginning to benefit from advances seen in recent years in NIRS hardware as well as in statistical and analytic approaches to fNIRS data. The migration to fiber-free, wearable, and high-channel-count devices are beginning to enable fNIRS and DOT approaches to provide a genuine alternative to fMRI. The first study to use wearable HD-DOT in an infant population was published in 2021.^288^ Meanwhile, researchers continue to explore new frontiers with fNIRS approaches, particularly in the use of increasingly naturalistic stimuli^289^^,^^290^ and applications in resource-poor settings.^291^^,^^292^

However, there are still significant challenges to address in functional neuroimaging in infants with diffuse optical methods. Devices with truly infant-optimized ergonomics remain rare, and the use of sub-optimal mechanical designs continues to limit the applicability and repeatability of optical measurements of the infant brain. Motion artifacts are another critical hurdle in the infant population. While artifact correction methods continue to improve,^123^^,^^124^ the field still lacks standardized methods that can be reliably and automatically applied to all types of diffuse optical data.

In the coming years, we can expect the dramatic improvements we have seen in optical devices that measure absolute tissue parameters at a single scalp location to coalesce with those of the wearable, high-density, wide-field-of-view devices that are now beginning to dominate functional optical neuroimaging. The result will be wearable devices that can be applied for extended periods and provide quantified, three-dimensional images of blood flow, tissue saturation, and metabolic activity in the infant brain at the cot-side and in real-time.

### 4.2 Developmental Disorders

Aside from providing valuable information on the cognitive processes during development, optical techniques have fostered novel studies to better understand how common disorders often diagnosed during childhood affect development through brain function. Here we highlight the clinical need as well as exciting developments in three such disorders: autism spectrum disorder, attention deficit disorder, and developmental coordination disorder.

#### 4.2.1 Autism spectrum disorder (ASD)

Autism spectrum disorder (ASD) is a serious psychiatric disorder defined by behavioral deficits in social functioning, communication, restricted interests, and repetitive behaviors.^293^^–^^295^ It is estimated that ASD affects 1/100 children worldwide.^296^ Early behavioral and educational interventions, starting at 18-24 months of age, improve outcomes in subsets of patients.^297^^,^^298^ Neuroimaging methods, including both task-based fMRI and task-free functional connectivity MRI, have demonstrated sensitivity to neural signatures of ASD that may inform diagnosis and track responses to interventions.^299^^,^^300^ However, the loud and constraining nature of MRI-based neuroimaging severely limits studies on direct within-room social communication, auditory processing, language generation, and overt motion. In addition, it presents an excessively challenging setting for sensitive participants, such as school-aged children and particularly young infants, toddlers, and those severely affected with ASD.^301^ Functional neuroimaging with diffuse optics is a potential solution to providing a more naturalistic functional neuroimaging environment that will be amenable to studies on children with ASD.

Multiple studies have shown progress in applying optical neuroimaging methods to patients with ASD,^302^^,^^303^ and recent developments in HD-DOT techniques have dramatically improved image quality, enabling fMRI-comparable image quality in an open, upright seated environment, even in low-resource settings.^4^^,^^304^^,^^305^ With optical methods, important and novel questions about ASD throughout development, including the effects of clinical interventions, can be addressed in a comfortable, free-moving, and naturalistic setting. The wearability and the open scanning environment of fNIRS and HD-DOT will also allow for motor tasks that are more challenging in the confining environment of an fMRI to be administered in the ASD population, e.g., studies of infants or toddlers in dyadic interaction and paradigms exploring joint attention-related phenomena. These studies may also provide a foundation for future investigations pairing genotyping with functional neuroimaging. Future wearable, fiber-less HD-DOT imaging^11^ may facilitate the identification of common mechanisms by which disparate genetic pathways to ASD result in the ASD phenotype.

#### 4.2.2 Attention deficient hyperactivity disorder (ADHD)

fNIRS also has a great potential for assessing children with attention deficit hyperactivity disorder (ADHD), for many of the same reasons outlined in Sec. 4.3.1. Regular fNIRS assessment with drop-out rates of less than 5%, even for ADHD children, are commonly observed owing to the modality’s ability to enable monitoring in naturalistic environments. In a series of recent studies, fNIRS has been used to determine how commonly-used treatments for ADHD – including atomoxetine,^306^^,^^307^ osmotic release oral system methylphenidate,^308^^,^^309^ and guanfacine extended release^310^—affect the neurocognitive functions of ADHD children during an inhibitory and attentional task. Specially, it has been shown that, unlike age- and gender-matched typically developing controls, pre-medicated ADHD children do not activate the right inferior and middle prefrontal gyri (IFG/MFG) during the inhibitory task. The lack of activation of right IFG/MFG was acutely normalized after administration of atomoxetine and methylphenidate, but not after placebo administration. Interestingly, the administration of guanfacine-extended release activated the right inferior parietal cortex (IPC), but not the right IFG/MFG. On the other hand, the attentional task recruited the right IFG/MFG and IPC in typically developing children. The right prefrontal activation was normalized after atomoxetine and methylphenidate administration in ADHD children, but the right IFG normalization was specific to atomoxetine. These results demonstrate that fNIRS is sensitive to the differential neuropharmacological effects of atomoxetine, methylphenidate, and guanfacine-extended release as they up-regulated the noradrenaline and dopamine systems in the inhibitory and attentional networks in the brains of ADHD children.

Interestingly, such differences in cortical activation patterns, which may reflect underlying neuropharmacological effects, seem to differ between subtypes of ADHD children, namely, medication-naïve ADHD children with or without ASD.^311^ During the inhibitory task, medication- naïve ADHD children without ASD exhibited reduced right prefrontal activation, but methylphenidate medication significantly elicited right prefrontal activation. However, medication-naïve ADHD children with ASD showed marginal activation before the first day’s medication and reduced activation after methylphenidate medication in the right prefrontal area. Thus, ADHD with and without ASD can be characterized by a different underlying neurofunctional pathology of inhibition control using fNIRS. In a typical clinical diagnosis, distinction between ADHD with or without ASD takes careful behavioral observation over six months, but the fNIRS-based monitoring would provide a quick but useful hint on the presence of ASD-comorbidity and help clinicians to formulate drug-treatment plans for the ADHD children.

#### 4.2.3 Developmental coordination disorder (DCD)

Developmental coordination disorder (DCD) is a neurodevelopmental condition characterized by deficits in acquiring and executing motor skills within the *Diagnostic and Statistical Manual* 5^th^ edition. DCD is often manifested in school age children as clumsiness and slowness, impacting personal, social, academic, and occupational functioning.^312^ Although there is currently no cure, early steps can be taken to reduce the emotional, physical, and social consequences associated with this disorder. However, identifying children with DCD is difficult as the etiology of DCD remains unclear. Previous studies using traditional neuroimaging under rigid, confined, and unrealistic conditions, resulting in limited understanding of the neural correlates of the motor-cognitive dysfunction.^313^

As the deficits in DCD are motor-related, mobile fNIRS is an ideal candidate tool, as it allows monitoring brain activity during gross motor movements. One recent study collected fNIRS during active ambulatory behavioral task execution from both typically developing and DCD children in one of the largest neuroimaging studies of DCD.^313^ Children performed both cognitive and physical tasks in both single and dual conditions. The study confirmed that DCD is a motor-cognitive disability, as gross motor complex tasks revealed dysfunction within the right middle and superior frontal gyri of the prefrontal cortex. By incorporating behavioral performance, decreased neural efficiency in these regions were revealed in children with DCD, specifically during motor tasks. Evaluating the disorder in such ecologically valid contexts could help optimize the interventions and open the door for precision therapies.

### 4.3 Cognitive Neuroscience Applications

#### 4.3.1 Neuroergonomics

Understanding brain function in real-world applications is the next frontier for diffuse optics in human neuroscience. Existing studies with traditional neuroimaging approaches have accumulated overwhelming knowledge but are limited in scope, *i.e.*, only in artificial lab settings and with simplified parametric tasks.^314^ Diffuse optical techniques can fill the current gap by providing a reliable tool to the emerging and new interdisciplinary field of neuroergonomics, which aims to investigate human brain function in the wild, during unrestricted real-world tasks in everyday life contexts, and its relationship to action, behavior, body, and environment.^315^^,^^316^

This paradigm shift opens exciting experimental opportunities in relevant fields not yet fully explored by other traditional neuroimaging techniques. The ultra-portable wearable and wireless NIRS devices already mentioned overcome limitations and constraints imposed by traditional neuroimaging tools on experimental protocols, data collection settings, and task conditions at the expense of ecological validity.^16^^,^^317^^–^^319^ Recent fNIRS studies demonstrate examples of experimental scenarios that were not feasible until recently, such as speaker-listener coupling,^320^^–^^322^ mental workload decoding in real-time during a flight over the clouds,^323^ or while walking outdoors.^324^^,^^325^ In addition, fNIRS can provide continuous and repeated measurement for longitudinal evaluation, and this can be used not only to assess but also to enhance the training/skill acquisition for complex tasks^326^ or to provide online real-time feedback as clinical intervention.^327^ There is a vast potential offered by continuous neural measures to reshape our understanding of the brain mechanisms and how we engage with the world. Neuroergonomic applications with NIRS technology may eventually be an integral part of the way we learn new skills, do our work, and entertain ourselves.

Despite the progress, there are still significant shortcomings that plague the full utilization of NIRS for neuroergonomic applications, including low-to-no sensitivity for deeper brain areas, low spatiotemporal resolution, and low SNR. Non-portable, room-based NIRS instruments could address some of the challenges with denser sensor arrays and tighter coupling of sensors, but mobile and newer ultra-mobile versions are more affected by these limitations.^328^ In the meantime, ongoing sensor and signal processing improvements make systems more portable, reliable, and affordable. Newer generation ultra-portable wearable NIRS systems are already positioned to go outside the lab for continuous measurements over longer periods and less constrained setups. These newer systems can allow more natural multimodal integration with EEG for faster time-resolution or neurostimulation for guided modulation of brain activity and enable adaptive computer/machine interaction. Improvements in artificial intelligence/machine learning approaches to recognize and label behavioral patterns will provide task context to brain signals for expanded and more automated analysis. Together with the capability for continuous interpersonal neural measures from fNIRS, these improvements may soon yield new means of cooperation, training, gaming, and work.

#### 4.3.2 Social interactions

Humans are profoundly social. Understanding the neurobiology that underlies social behaviors is a frontier in social neuroscience.^329^^,^^330^ Conventional investigations of the neural underpinnings for social behaviors are based on single brain investigations. These conventions have led to a long-standing experimental paucity of two-person interactive experimental paradigms to investigate social behaviors. However, recent developments in fNIRS for hyperscanning (i.e., simultaneous brain scanning of two or more individuals during live interactions) pave the way for long-awaited studies of live interactions between individuals.^331^ Technical advances in fNIRS (see Secs. 2 and 3) and the growing importance of understanding the biological components of live and interactive human social behaviors have supported the emergence of dyadic neuroscience.^332^^–^^334^

From a Dyadic Neuroscience perspective, a single brain is only one half of a dynamic social unit.^335^^–^^337^ In the case of a dyad, pairs of individuals are “linked” together by meaningful and reciprocal exchanges of information.^338^^,^^339^ Quantification of dyadic properties includes measures of neural coupling, sometimes referred to as inter-brain synchrony or cross-brain coherence,^340^^–^^342^ which quantifies the extent to which the temporal patterns of neural signals across brains are correlated.^321^^,^^343^^–^^345^ The main questions include: What are these dyadic mechanisms? How can they be isolated? How can they be modulated in health and disease?

Multimodal acquisitions of many simultaneous measures, including EEG, face recognition, auditory recordings, eye-tracking, and physiological variables, enhance interpretations of fNIRS findings.^346^^–^^348^ Wearable systems with full-head and high-density spatial resolution promise continued advances. A vast array of development opportunities exists in potential fusions of collaborating disciplines. However, the absence of sensitivity in subcortical layers of the brain is a significant limitation, but it is balanced with the advantages of probing neural processes during live and natural social interactions. These advantages bring social neuroscience closer to applications that benefit ordinary lives and patient care.^349^

#### 4.3.3 Learning and education

Since NIRS is considerably more flexible regarding head motion tolerance and environmental conditions, it has become an attractive neuroscientific tool for investigating learning in real educational contexts. Moreover, some portable devices are also available, making it possible to collect the data at school, *i.e.*, a natural learning environment for the child. In addition, experiments with children usually demand the preparation be short so the participants are more collaborative during the acquisition. Thus, the ease of cap/band/net preparation in fNIRS is also helpful. 

The main progress in fNIRS applications in education is the exploration of interpersonal interactions during learning using hyperscanning.^322^^,^^350^ These situations connect with pedagogical constructs such as Vygotsky’s proximal development zone. Moreover, combining fNIRS signals and machine learning for mental states decoding made it possible to monitor and quantify attention, engagement, and learning.^351^

Current limitations in this field may also be seen as opportunities for technological development. In comparison to biomedical research, budgets in education are very limited. Thus, the availability of low-cost portable quality systems^352^ is crucial for impacting elementary schools. Furthermore, considering the specificities of educational contexts in which block or event-related designs are not feasible, novel experimental designs and the respective analytical methods are necessary. In the next five years, we can expect that fNIRS in education will go beyond the frontiers of academic research. Neurotechnology startups focused on education will provide hardware and analytical products to quantify engagement and learning. This innovation will allow experimental sciences to be more relevant in educational strategies and instructional resources.

#### 4.3.4 Perceptual-cognitive development

The advances in hardware and data analysis mentioned in the previous sections have enabled novel perceptual and cognitive development studies. Here, we highlight a number of exciting avenues of research that have recently emerged and that we think will be highly influential in fNIRS studies of perceptual-cognitive development in the next five years.

As caregivers and the environment are important in infants’ perceptual-cognitive development and its disorders, the use of fNIRS in social contexts and in interaction with caregivers is promising for revealing mechanisms of development and for understanding risk and resilience. To this end, hyperscanning allows researchers to assess how infants interact with and learn from their environment, such as within the mother–child dyad.^353^ These studies highlight that during real-life communication, infants’ brains and behaviors both shape and reflect those of adults.^354^

Another way in which fNIRS is supporting more ecologically-valid research in perceptual-cognitive development is through its application in a range of global health research projects.^292^^,^^355^^,^^356^ These projects are opening new horizons for understanding environment-specific impacts on brain development as well as increasing the diversity of research participants and the communities who are represented in studies of perceptual-cognitive development.^357^^,^^358^

Finally, multi-modality recordings (e.g., EEG with NIRS) are on the rise^359^^–^^361^ and in the next five years will provide a critical cross-modal comparison of the physiological processes underlying infants’ perception and cognition. Indeed, NIRS is uniquely poised to allow for these kinds of multimodal recordings as it does not have the same constraints as the MR environment.

## 5 Clinical Applications of Optical Spectroscopy and Imaging

As highlighted in a recent report from the Global Burden of Diseases, neurological disorders and injuries are the leading cause of disability and the second leading cause of death worldwide.^362^^,^^363^ Non-invasive optical methods have the potential to significantly contribute to the diagnosis, prognosis, or both situations in a wide array of these conditions. The wearability, high temporal resolution acquisition, and low-cost benefits of hardware developments discussed in Sec. 2 promise to permit continuous bedside monitoring, and individualized care can be envisioned with real-time data analysis using techniques outlined in Sec. 3. In fact, long-established continuous-wave cerebral oximetry approaches^162^ have already benefitted from some of these advances to track trends in cerebral oxygen saturation in the clinic. These commercially available, FDA-approved systems are now standard-of-care in several clinical applications, including surgical monitoring. Meanwhile, the surge of interest in DCS methods and their integration with increasingly small-footprint frequency-domain, time-domain, and broadband NIRS instruments has resulted in several novel cerebral monitoring devices that permit simultaneous assessment of CBF, absolute oxygen saturation, and cerebral oxygen metabolism consumption at the bedside.^96^^,^^364^ Moreover, mobile neurotechnologies demonstrate exceptional potential for decoding brain health and performance-related information. This section provides a broad overview of potential niches for such novel optical spectroscopy/imaging techniques in different clinical settings and discusses the assessment of several novel biomarkers of brain health that can be directly derived from these optically-obtained signals.

### 5.1 Outpatient Surveillance of Disease Progression and Prognosis

The non-invasive and low-cost nature of diffuse optical techniques lends itself well to the outpatient setting. Of particular interest for outpatient brain monitoring with diffuse optics are forms of intracranial cerebrovascular diseases (e.g., internal carotid artery disease, Moyamoya, sickle cell disease, etc.). Patients with these conditions are often at elevated risk for stroke and neurodegeneration as progressive cerebrovascular dysfunction leaves the tissue susceptible to ischemic and/or hypoxic injury. As such, the hemodynamic measurements quantified by NIRS and DCS have the potential to provide biomarkers of both disease severity and prognosis. Just like the glucose monitor for diabetes, the development of wearable, optics-based monitors of cerebrovascular health in patients with cerebrovascular disease could be pivotal to alert clinicians of times of hemodynamic stress that could result in brain injury/degeneration, thereby enabling rapid initiation of interventions aimed at restoring cerebral hemodynamics or at least minimizing perfusion deficits. For example, in patients with internal carotid artery disease (ICAD), the carotid arteries are highly prone to developing atherosclerotic plaque, which is a known risk factor for stroke.^365^^,^^366^ In these patients, NIRS and DCS have been used to demonstrate reduced cerebrovascular reserve and increased cerebral oxygen extraction fraction compared to controls.^367^^–^^372^ Moreover, the optically-measured parameters correlate with the current clinically-accepted measurement of vasomotor reactivity from transcranial Doppler ultrasound (TCD) both in the affected and nonaffected hemispheres.^370^ Importantly, optical-based measures have been associated with the severity of the stenosis,^368^^,^^372^ reinforcing the potential of NIRS/DCS to follow ICAD progression and prognosis of neurodegeneration induced by this disease.

### 5.2 Global Health

In the past 30 years, the number of individuals affected by neurological diseases has risen substantially, particularly in low-income and middle-income countries (LMICs).^362^^,^^363^ Moreover, mental health disorders, including depression, anxiety, and schizophrenia, affect about one-third of the global population across the lifespan^373^ and are associated with ten years shorter life expectancy.^374^ The lack of neurodiagnostic capacity contributes to neurologic disorders’ high morbidity or mortality in lower-income settings. In addition to the absolute barrier of unavailability of neurodiagnostic tests, many people in LMICs face long wait times and low affordability, with women less likely to receive appropriate diagnosis and treatment.^375^ These reports demonstrate the urgent need for viable and affordable neurodiagnostic tools in LMIC, and NIRS and DCS can play this role, being low cost, easy to use, and feasible in ambulatory settings.

Few studies, mostly in children—where NIRS and DCS have provided more robust measures—have shown the feasibility of using these modalities in a low-resource setting. A pilot study at the Cure Hospital in Uganda showed that a postoperative decrease in cerebral tissue optical scattering (measured by frequency-domain NIRS) was highly predictive of treatment failure in infants undergoing endoscopic third ventriculostomy combined with choroid plexus cauterization to treat post-infectious hydrocephalus.^376^ Early detection of treatment failure is critical in LMIC because most patients do not have easy access to medical care once they leave the hospital. In a nutritional intervention study in Guinea Bissau, researchers found increases in ipsilateral CBF (measured with DCS) in children who had taken a new dietary supplement for 23 weeks compared to controls.^377^ This study illustrates how DCS can objectively measure the supplement’s impact on brain growth. When paired with other fNIRS studies, they demonstrate improved cognitive performances when trying to ameliorate the effects of malnutrition on brain development. On this front, several studies have been conducted in Africa and Asia to investigate brain development delays in resource-poor countries.^291^^,^^292^^,^^305^^,^^356^^,^^378^^–^^380^ While the use of NIRS and DCS show promises in children, more studies in adults living in low-resource settings need to be carried out to show the diagnostic capabilities of optical methods.

### 5.3 Critical Care Management

In neurocritical care, clinical management focuses largely on ensuring adequate oxygen delivery to the tissue to prevent irreversible cell death. In severe cases—such as severe traumatic brain injury (TBI), hemorrhagic stroke, or subarachnoid hemorrhage (SAH)—an invasive sensor will be placed in the parenchyma or ventricles to directly monitor oxygen tension, blood flow, and/or ICP. In the absence of this invasive monitoring, which is often not warranted due to the risks associated with implantation and infection, there is a general lack of bedside tools to monitor brain function. Thus, it is of no surprise that non-invasive local measurements of oxygenation and CBF with NIRS and/or DCS techniques, respectively, have been the subject of a plethora of research studies in neurocritical care patients with the hopes of providing valuable information about injury severity and progressions. Diffuse optical devices have been shown to detect hypoxia^381^^,^^382^ and inadequate CBF,^383^^,^^384^ which is relevant for tracking patient status and outcomes. For example, in neonates with hypoxic-ischemic encephalopathy, the metabolic response to hypoxic episodes measured with bNIRS was shown to be associated with outcome.^385^ In SAH, pilot work suggests diffuse optics can detect cerebral vasospasm with higher sensitivity than TCD;^386^^–^^388^ recent data suggest that the microvascular CBF response measured with DCS to a pharmacological intervention intended to treat vasospasm is associated with development of delayed cerebral ischemia.^389^ In ischemic stroke, studies have revealed one in five patients exhibit a blunted or paradoxical cerebral blood flow response to an orthostatic challenge, a common clinical approach to optimize cerebral perfusion after injury.^390^^–^^392^ More recently, new optical devices have allowed the monitoring of novel therapeutic approaches intended to increase blood flow and oxygenation in the ischemic penumbra.^393^^–^^398^ Moreover, exciting new work suggests optics may be sensitive to ICP. This growing area of research is discussed more in depth in Sec. 5.7.

Beyond the critical care unit, optical monitoring may also play a role in acute treatment settings as the dreams of mobile neuromonitoring become a reality. For example, a new handheld NIRS-based medical screening tool has recently been employed to detect brain bleeding and assess brain injury at the site of an accident, in the ambulance, and repeatedly within the hospital – something that could previously only be done in hospital settings with bulky equipment.^399^ This new generation mobile NIRS system is *de novo* FDA approved, deployed in 42 countries on six continents in civilian and military hospitals and has become the standard of care for children and for sports medicine in some European countries. Another promising application for emergent monitoring is in clinical guidance during and after cardiopulmonary resuscitation (CPR). Several studies have demonstrated that optical brain monitoring during CPR is feasible,^400^^–^^405^ and recent work suggests that brain-based CPR optimization promises to enhance neurologic outcomes. In a swine model, successful resuscitation was predicted by FD-NIRS neuromonitoring.^405^ Encouraging associations between NIRS measures and outcomes have also been observed.^400^^–^^403^ After arrest, pilot studies have explored using NIRS to individualize blood pressure targets^406^^,^^407^ as well as the use of DCS measures of CBF low-frequency power^408^ and pulsatility as a neurologic biomarker.^409^

Optical imaging also promises to provide valuable information in epilepsy monitoring. Previous studies have explored CW-NIRS to measure the hemodynamic signatures of epileptic events such as seizures^410^^–^^414^ and interictal epileptiform discharges.^415^^,^^416^ Notably, NIRS allows practical assessment of epilepsy in neonates^417^^–^^419^ and children.^420^^–^^424^ Overall, a multimodal approach with EEG and optical techniques has resulted in improved localization of brain regions that generate abnormal activity. These findings pave the way to study several aspects of epilepsy, ranging from regional differences of epileptic onsets to longitudinal monitoring of drug-resistant epilepsy.

### 5.4 Surgical Monitoring

Management of cerebral hemodynamics in surgical rooms is another critical area in which optical imaging has the potential for significant impact in patient outcomes. Applications range from guiding neurovascular interventions that prevent or acutely treat stroke, to assessing pain, to ensuring adequate cerebral perfusion during cardiac surgery, to avoiding ischemia during spinal cord surgeries. For example, NIRS/DCS intraoperative monitoring of cerebral hemodynamics during carotid endarterectomy has resulted in significantly faster and more sensitive detection of cerebral hypoperfusion induced by temporarily clamping the internal carotid artery during surgery compared to current gold standard methods.^425^^–^^427^ In addition, optical measurements can be predictive of cerebral hyperperfusion syndrome (a rare but critical complication in ICAD patients who undergo surgery) after both carotid endarterectomy^428^ and carotid artery stenting.^429^ Similarly, NIRS/DCS has the potential to provide valuable prognostic information during recanalization induced by mechanical thrombectomy in acute stroke patients.^394^^,^^398^ Other emerging applications include optical guidance of extracorporeal perfusion to optimize brain health during cardiac surgery and critical care.^430^^–^^435^ For example, in aortic arch repair surgeries involving hypothermic cardiopulmonary bypass and selective antegrade cerebral perfusion, optics can accurately characterize the suppression of cerebral oxygen metabolism^436^ and whether oxygen supply meets demand.^437^ The degree of metabolic suppression was recently associated with white matter injury in neonates undergoing deep hypothermic circulatory arrest.^435^ Optical oxygen delivery and autoregulation metrics may also individualize extracorporeal perfusion management.^432^^,^^433^^,^^438^^–^^441^ In a related vein, modifications in diffuse optical hardware have accelerated the use of NIRS and DCS to monitor local spinal cord hemodynamics intraoperatively. This approach has been tested in ovine^442^^–^^444^ and porcine^445^^,^^446^ models so far, showing the feasibility and safety of using optical spectroscopy to monitor different sites of the spinal cord and identify early ischemia that can result in paralysis and paraparesis.^442^^,^^446^^,^^447^

Overall, the exciting results outlined in Secs. 5.1–5.4 as well as countless other examples suggest that optics-based neuromonitoring can significantly impact patient care in a wide range of clinical settings. In the near future, we anticipate large scale, multi-center studies will pave the way towards integrating diffuse optics into standard clinical practice. Moreover, we envision that emerging artificial intelligence approaches will be employed to further enhance the usefulness of optical data by personalizing patient care.

### 5.5 Brain-Computer Interface for Rehabilitation

A brain-computer interface (BCI) is a communication system that allows its users to control computers and other external devices using brain activity.^448^ fNIRS-based BCI has gained much attention in the research community due to its recent use as a non-invasive BCI neurorehabilitation tool to improve cognitive and motor performance.^449^ This approach to active rehabilitation aims to replace the disrupted neuromuscular pathways of humans caused by disorders or amputation with wearable robotic devices.^450^ The recent advances in fNIRS-based BCI studies focus on their applications for (i) the movement control of robots and exoskeletons, (ii) detecting and preventing brain disorders, (iii) analyzing and controlling psychophysiological states, and (iv) monitoring and controlling pathological and normal cognitive activity.^451^

Despite an exponential increase in fNIRS-based BCI-related publications and financial investment efforts during the last two decades, the clinical success of fNIRS-based BCI applications is still pending.^452^ The research has been chiefly technical and methodological. The major limitation of the current fNIRS is its binary nature in mental communication, as it can accurately record the answers to simple yes/no questions, but the classification accuracy is compromised when more complicated responses are involved.^453^^,^^454^ Another challenge with fNIRS as a BCI is the delay in the hemodynamic response, which hinders real-time communication.^27^ However, the initial dip and fast optical response detections in recent studies have shown a faster information transfer rate.^455^

The future of fNIRS occupies a significant space within neuroscience, particularly in real-world cognition, neurodevelopment, and social interaction. There is much room for fNIRS-based BCI research, particularly in its applications, e.g., hyperscanning methods that simultaneously study multiple participants’ brain activity in social interactions.^456^ The future of portable, non-invasive, and wearable fNIRS-based BCIs for neurorehabilitation and neurofeedback applications lies with the use of hybrid EEG-NIRS systems and bundled-type NIRS probes and the detection of the initial dip and improved sampling rate.^457^^,^^458^ The availability of wearable NIRS devices has paved the way for new and revolutionary neuroimaging investigations that might grow over the following years, such as wearable high-density systems for studies in naturalistic settings.^459^

### 5.6 Biomarkers of Vascular Health

The brain is uniquely sensitive to ischemic injury from inadequate flow and is also vulnerable to injury from hyperemic states. Thus, multiple layers of neuro-hormonal and vascular responses tightly regulate cerebral blood flow to ensure the local supply is commensurate with metabolic demand. Two of these regulating mechanisms, cerebral autoregulation and cerebrovascular reactivity, are becoming more commonly monitored with diffuse optics. We discuss these parameters and their future outlook in the sections below.

#### 5.6.1 Cerebral autoregulation

Cerebral autoregulation refers to the constraint of blood flow in the brain in the presence of changes in blood pressure. This process is mediated by vessel reactivity, and it acts to prevent potentially harmful fluctuations in cerebral blood flow. When arterial blood pressure decreases from normal levels, arteries and arterioles within the brain dilate to decrease vascular resistance and maintain CBF at normal levels. However, when arterial blood pressure is reduced to a degree whereby the vasodilatory response is exhausted, CBF decreases, and the brain is at risk for ischemia. This transition point is called the lower limit of autoregulation, and it serves as a clinically significant reference point for arterial blood pressure management. Unfortunately, because there is no approved method to delineate this lower limit in clinical practice, and because it can vary between subjects and across disease states, clinical care of conditions that impair cerebral perfusion pressure and are associated with ischemic injuries—such as head trauma, subarachnoid hemorrhage, sepsis, and cardiac surgery—involves management of arterial blood pressure in ignorance of this lower limit.

Many published methods exist to measure autoregulation.^460^ All methods are composed of three essential elements: 1) a measure of perfusion pressure (such as arterial blood pressure or cerebral perfusion pressure), 2) a measure of a cerebral vascular property (such as blood flow, blood volume, or blood oxygenation), and 3) a measure of the relatedness of the cerebral vasculature to the perfusion pressure (such as correlation, coherence, phase shift or gain of transfer). The modalities employed and the permutations of combinations of these three elements are numerous. Given the numerous advantageous attributes of NIRS and DCS discussed herein, including their low-cost and bedside monitoring capabilities, these tools hold promise to measure cerebral vascular properties (element 2). Indeed, literature reports of autoregulation assessment with commercially available CW-NIRS systems abound, and more recently, assessment with DCS measurements of blood flow has become more prevalent.^388^^,^^432^^,^^461^^–^^463^ While DCS provides direct measurements of CBF, NIRS measurements of blood volume, cerebral oxygenation, or the difference between the concentrations of oxy- and deoxy-hemoglobin have also been used as surrogates of CBF. Dynamic relationships between the optical measurements of CBF (or its surrogate) and mean arterial pressure (MAP), are then examined to yield measures of cerebral autoregulation. Most commonly, these dynamic relationships are determined by transfer function analysis or correlation analysis. In contrast to TCD assessments of cerebral autoregulation that rely on measurements of CBF velocity in the middle cerebral artery,^464^^,^^465^ a significant feature NIRS/DCS is that the tools provide complementary local measurements of the autoregulatory capacity at the microcirculation level. Moreover, the local measurements made with NIRS and DCS lend themselves well to spatially resolved or imaging implementations.

The main limitation of autoregulation monitoring is the need for oscillations in perfusion pressure at relatively low frequencies. Pulse and respiratory frequency oscillations are too fast to reliably engage the autoregulation mechanism, which has an upper frequency limit of 0.10 – 0.15 Hz.^466^ Transients and oscillations at lower frequencies (i.e., 0.008 – 0.03 Hz) are ideal for monitoring autoregulation, but these phenomena are often irregular. The lack of reliable low-frequency blood pressure oscillations creates random noise in the autoregulation analysis, so time windows used for assessment must be averaged together or excluded. Thus, autoregulation monitoring can be slow, especially when the arterial blood pressure is static. Furthermore, there is inadequate clinical experience with autoregulation monitoring outside of the research arena to know if widespread application will be feasible or safe. Removal of statistical noise using appropriate signal processing will improve the safety of this modality,^467^ but proper phase II studies of safety are needed to demonstrate a lack of harmful side effects from corrective interventions based on autoregulation monitoring. The application of autoregulation monitoring will likely lead to clinical management that utilizes higher arterial blood pressure targets for most conditions and possibly that tolerates lower arterial blood pressure management for other conditions. The safety of this practice change must be determined before widespread use occurs.

Anticipated advancements in autoregulation monitoring include: 1) improved SNR to render precise and rapid delineation of the lower limit of autoregulation; 2) improvement in the clinical user interface such that automated interpretation renders easy-to-read guardrails for arterial blood pressure management without needing to be an expert in the mechanics of autoregulation monitoring; 3) studies demonstrating feasibility (reduction of the burden of hypotension below the lower limit), safety (does optimizing blood pressure to the brain harm other organs?), and efficacy (a reduction in patient morbidity with the application of autoregulation monitoring). Diffuse optical methods such as DCS and NIRS offer a good option for autoregulation assessment and monitoring in that they are non-invasive, portable, safe, and offer a strong sensitivity to local microvascular flow. The first general challenge mentioned above, namely the achievement of a high SNR also implies a suitable suppression of confounding contributions from extracerebral tissue hemodynamics and is currently being actively explored in light of the many research advances that have been described in this article. The second and third challenges mentioned above require clinical validation studies and technological refinements in the user interface that are also being explored in the field.

#### 5.6.2 Cerebrovascular reactivity

Cerebrovascular reactivity (CVR) describes the process why which the cerebral vasculature dilates or constricts in response to a vasoactive stimulus like dissolved oxygen or carbon dioxide. CVR is an integral mechanism in brain homeostasis. Unfortunately, this mechanism can become damaged in a wide range of conditions, including cerebrovascular disease,^468^^–^^471^ stroke,^472^^–^^475^ cardiac arrest,^476^ and traumatic brain injury.^477^^–^^480^ Moreover, several studies have suggested that impaired CVR may serve as a prognostic biomarker of functional outcome.^481^^–^^484^ For example, impaired CVR has been shown to be a more robust predictor of poor neurologic outcome than standard imaging-based scoring systems in patients with carotid artery stenosis.^473^ Thus, bedside quantification of CVR could serve a valuable role in guiding patient care.

Assessment of CVR requires both a vasoactive stimulus (either by spontaneous breath-holding or through administration of acetazolamide or carbon dioxide) as well as the quantification of the cerebral blood flow response to the stimulus. The latter requirement is well suited for either DCS measurements of CBF, or NIRS measures of blood flow surrogates like cerebral blood volume or oxygen saturation. Indeed, both NIRS and DCS have been used to quantify CVR in a range of pilot patient cohorts, including children with congenital heart defects^485^^–^^487^ and obstructive sleep apnea,^488^ adults with cerebrovascular disease,^368^^,^^369^^,^^489^^,^^490^ as well as healthy controls.^189^ However, the main limitations of assessing CVR with diffuse optics are the poor spatial sensitivity, depth sensitivity limited to superficial cortex, and contributions of extracerebral hemodynamics. Spatial and depth sensitivities are most relevant when regionally heterogenous CVR is thought to be present (e.g., in the case of ischemic stroke); however, this limitation is less relevant in patient cohorts wherein global impairments in CVR are commonly observed (e.g., traumatic brain injury, sickle cell disease). Extracerebral contributions to the diffuse optical signal are of concern when assessing CVR, as the stimuli utilized to elicit the CVR response often also elicit systemic changes (i.e., increase in blood pressure and/or heart rate) that influence both scalp and brain perfusion. These systemic changes can confound the estimation of CVR in a manner that is not present with other perfusion modalities.

In the future, we anticipate a growing focus on assessment of CVR with diffuse optics as the recognition of the importance of this biomarker of vascular health becomes more widespread. Towards this end, researchers will need to capitalize on both hardware and software advancements discussed in previous sections to enhance depth penetration and spatial coverage of the measurements, as well as to provide real-time assessments of CVR at the bedside. Moreover, we will need a better understanding of the reproducibility of these measurements, including not just measurement repeatability, but also the sensitivity of CVR to factors such as time of day, caffeine intake, hormone status, age, and sex. Finally, given the relative ease of CVR assessment with diffuse optics (compared to MRI, PET, or even TCD), the field could benefit from the continued development of more tolerable and practical means of applying a vasoactive stimulus that do not require inhalation of ${\mathrm{CO}}_{2}$ gas or intravenous administration of acetazolamide. Recent work that quantifies the CBF response to fluctuations in end-tidal ${\mathrm{CO}}_{2}$ that occur naturally during free-breathing while at rest or due to subject-guided breath modulation^491^^,^^492^ would provide a complementary, minimally burdensome means to assess CVR with diffuse optics.

### 5.7 Optical Measurements of Intracranial Pressure (ICP)

Intracranial pressure (ICP) is an important parameter of cerebral health in various diseases. Prolonged elevation of ICP, or intracranial hypertension, is a significant cause of brain injury in numerous maladies.^493^^–^^495^ The measurement of ICP can aid in the rapid diagnosis of intracranial hypertension, and the regulation thereof with external ventricular drains can prevent adverse consequences, including malperfusion of the brain tissue leading to ischemia.^496^ Indeed, regulating the level of ICP when it exceeds normal bounds has been demonstrated to improve patient outcomes from brain injuries, including TBI^497^^–^^500^ and hydrocephalus.^501^ Currently, clinical measurements of ICP are invasive, with pressure transducers placed into the parenchyma, ventricles, or just below the skull.^502^^,^^503^ The invasiveness of these measurements limits their use to the most critically ill patients, wherein the benefit of ICP monitoring outweighs the risk of transducer placement. While non-invasive alternatives to measuring ICP have been proposed using a variety of approaches,^504^^,^^505^ none have garnered widespread adoption to date.

Proof-of-concept studies suggest that diffuse optical methods hold promise to relate microvascular hemodynamic changes (i.e., either hemoglobin concentration changes with NIRS or cerebral blood flow changes with DCS) to intracranial pressure. For example, changes in oxy- and deoxy-hemoglobin have qualitatively been shown to correlate with ICP,^506^ and a transfer function approach has been used to relate slow changes in oxygenated hemoglobin concentration to changes in ICP.^507^ More recently, quantitative estimation of ICP has been demonstrated using non-invasive DCS measurements of CBF waveform morphology combined with machine learning. These approaches have yielded excellent accuracy of <4  mmHg in an animal model^508^ and clinical populations of benign enlargement of subarachnoid spaces and traumatic brain injury.^509^

Intracranial hypertension has also been assessed via measurement of the cerebral critical closing pressure (CrCP), the isotropic pressure compressing the cerebral arterioles.^510^^–^^513^ CrCP depends on both ICP and vasomotor tone, and the difference of mean arterial pressure and CrCP is the cerebral perfusion pressure that drives CBF. Given the dependence of CrCP on ICP, elevated CrCP is a promising biomarker of intracranial hypertension. Recent work has demonstrated that the estimation of CrCP using DCS measurements of pulsatile blood flow shows a good correlation with CrCP estimated with TCD measurements of blood flow velocity in the large arteries of both healthy subjects^513^ and stroke patients.^345^ Moreover, an association between DCS-measured CrCP and intracranial hypertension has been shown in infants with hydrocephalus.^514^

In general, noninvasive ICP and CrCP estimation is challenging due to the complex interplay of parameters such as cerebral blood volume, arterial pressure, vascular tone, and compliance, which are usually unknown during the measurement.^515^ The dynamic relationship between these variables is further complicated by the pathologies inherent to individual disease states, making results difficult to interpret clinically.^516^ To address these challenges, continuing the current two-pronged strategy of implementing both data- and model-driven approaches are warranted in further clinical validation studies. As progress in these areas continues, NIRS- and DCS-based ICP and CrCP extraction methods could enable continuous, non-invasive assessment at the bedside, allowing for brain pressure monitoring to be extended to cohorts for whom placing an invasive sensor is not possible or contraindicated.

## 6 Final Remarks

This status report aims to highlight the abundant recent advances of the field of optical imaging and spectroscopy in the human brain, as well as to provide an outlook for the next five years. Over the past five to ten years, we have seen the emergence of a plethora of new approaches to quantify the optical properties and blood flow dynamics of deep tissue more accurately and with a higher signal-to-noise ratio. Meanwhile, existing techniques have benefited from optoelectronic advances that have led to smaller, faster, and more sensitive data acquisition. The design of denser, more compact, and portable instruments is already a reality and a target for dissemination in the near future. Simultaneously, data analysis methods have leveraged the power of diffuse optical instruments by providing novel approaches that lead to more clear interpretation of the data acquired. Each of the technologies and methods highlighted in this report has advanced based on the collaborative and complementary efforts of a wide range of experts who have been inspired and motivated by the higher demand from a growing community of researchers and users. As we embark on the future of our growing field, harnessing the power of collaboration across different research areas and laboratories will strengthen the field and accelerate pioneering initiatives targeting noninvasive optical imaging of the brain.

While diffuse optics has been viewed by some as exclusively as a side or complementary technique to other neuroimaging approaches in the past, Secs. 4 and 5 demonstrate that the scenario is rapidly changing, driven in part by the impressive hardware and software advances highlighted in Secs. 2 and 3. Over the past decade, NIRS has opened new and exciting directions in numerous research areas, including developmental, cognitive, and social neuroscience. In the clinic, NIRS and its combination with DCS have provided means to noninvasively expand our knowledge about other aspects of cerebral physiology, both at the patient’s bedside and outside healthcare facilities. Undoubtedly, the use of diffuse optical techniques has resulted in novel insights into a wide variety of clinical conditions that affect millions worldwide. And it is worth noting that the topics covered in Secs. 4 and 5 are just a glimpse of the current applications, biased toward our own research interests, and not a comprehensive list of all opportunities for optical imaging in neuroscience and the clinic.

Despite all the recent groundbreaking progress reported herein, the most exciting news is that we seem to be only at the beginning of this optical revolution. The previous sections attempted to identify critical bottlenecks and point future directions for diffuse optics to keep advancing. There are substantial challenges ahead, which also can be seen as unique opportunities for students and early-career researchers to jump in and enjoy what we envision as a bright future for diffuse optics in neuroscience and clinical applications!
